# The Activity of Gold Nanoparticles Synthesized Using *Helichrysum odoratissimum* Against *Cutibacterium acnes* Biofilms

**DOI:** 10.3389/fcell.2021.675064

**Published:** 2021-09-13

**Authors:** Marco Nuno De Canha, Velaphi Clement Thipe, Kattesh V. Katti, Vusani Mandiwana, Michel Lonji Kalombo, Suprakas Sinha Ray, Rirhandzu Rikhotso, Arno Janse van Vuuren, Namrita Lall

**Affiliations:** ^1^Department of Plant and Soil Sciences, University of Pretoria, Pretoria, South Africa; ^2^Department of Radiology, Institute of Green Nanotechnology, University of Missouri Columbia, Columbia, MO, United States; ^3^Chemical Cluster, Centre for Nanostructures and Advanced Materials, Council for Scientific and Industrial Research, Pretoria, South Africa; ^4^DST/CSIR National Centre for Nanostructured Materials, Council for Scientific and Industrial Research, Pretoria, South Africa; ^5^Centre for High Resolution Transmission Electron Microscopy, Nelson Mandela University, Port Elizabeth, South Africa; ^6^School of Natural Resources, University of Missouri, Columbia, MO, United States; ^7^College of Pharmacy, JSS Academy of Higher Education and Research, Mysuru, India

**Keywords:** *Cutibacterium acnes*, *Helichrysum odoratissimum*, gold nanoparticles, bacterial adhesion, biofilm

## Abstract

The human skin is home to millions of bacteria, fungi, and viruses which form part of a unique microbiome. Commensal microbes, including *Cutibacterium acnes* can occasionally become opportunistic resulting in the onset of dermatological diseases such as acne. Acne is defined as a chronic inflammatory disorder based on its ability to persist for long periods throughout an individual’s life. The synthesis of gold nanoparticles (AuNPs) was performed using the bottom-up approach by reduction of a gold salt (HAuCl_4_.3H_2_O) by the methanol extract (HO-MeOH) and aqueous decoction prepared from the dried aerial parts of *Helichrysum odoratissimum* (HO-Powder). The HO-MeOH and HO-Powder AuNPs were prepared as unstabilised (−GA) or stabilized (+GA) by the omission or addition of Gum Arabic (GA) as the capping agent. The characterization of the AuNPs was performed using Transmission Electron Microscopy (TEM), dynamic light scattering (DLS), Ultraviolet-Visual spectroscopy (UV-Vis), Thermogravimetric Analysis (TGA), X-Ray Diffraction (XRD) and Zeta-potential. The MBIC_50_ values for HO-MeOH − GA and HO-MeOH + GA were 1.79 ± 0.78% v/v and 0.22 ± 0.16% v/v, respectively. The HO-Powder AuNPs showed potent inhibition of *C. acnes* cell adhesion to the 96-well plates. The HO-MeOH − GA and HO-Powder + GA exhibited IC_50_ of 22.01 ± 6.13% v/v and 11.78 ± 1.78% v/v, respectively. The activity of the AuNPs validated the anti-adhesion activity of the methanol extract in the crude form. The study emphasizes the selectivity of *H. odoratissimum* AuNPs for the prevention of *C. acnes* cell adhesion and not antimicrobial activity, which may prevent the emergence of resistant strains of *C. acnes* through reduced bactericidal or bacteriostatic activity, while targeting mechanisms of pathogenesis.

## Introduction

The human skin is a host to numerous species of bacteria, fungi, and viruses; each with their own optimal micro-environment. Differences in skin physiology, including oil content, moisture content or absence thereof, have a large influence on the variability of microbial species. These microorganisms have essential functions as protectors against invasive pathogens, inducers of immune responses, and metabolizers of natural products. In addition to the protective role of commensal microbial species, the human skin provides a physical barrier to potential pathogens. Factors influencing the onset of skin disease and even systemic disease often include damage to the skin which affects barrier protection and an imbalance between beneficial host microorganisms and invasive pathogenic microorganisms (Byrd et al., 2018). *Cutibacterium acnes* is a Gram-positive, anaerobic rod which is considered one of the major species that inhabits the human skin and mucosal surfaces. Although this microorganism has been described as a commensal, it has been implicated in the progression of the inflammatory skin disorder acne vulgaris (acne) and other infections related to orthopedic prosthetic devices and prostatitis, suggesting that it is more likely an opportunistic bacterium (McDowell et al., 2013; Sahdo et al., 2013). Acne is defined as a chronic inflammatory disorder based on its ability to persist for long periods in an individual’s life and its ability to recur often resulting in relapse (Tuchayi et al., 2015). The word cosmeceutical refers to a cosmetic product that contains active constituents which promote health through the induction of drug-like benefits. Natural ingredients, especially those derived from plants, have been used for hundreds of years to promote skin health and their use is becoming more popular, with ingredients of modern cosmetic formulations often containing these ingredients as the active constituents (Ribeiro et al., 2015). South Africa is home to 24,000 higher plant species of which approximately 3,000 species have been reported to have therapeutic or medicinal properties (De Wet et al., 2013). The genus *Helichrysum* is reported consist of around 500 species, of which 245 are indigenous to South Africa. Species from this genus are used across Africa and Europe in the treatment of wounds, infections and skin disorders such as atopic dermatitis (Lourens et al., 2004). *Helichrysum odoratissimum* commonly referred to as the most fragrant Helichrysum or impepho, is an aromatic perennial shrub which is not only widely distributed throughout South Africa but can also be found in Lesotho, Malawi, Mozambique, Swaziland, and Zimbabwe. Traditionally the plant is boiled and used as a facial ointment for pimples (Swelankomo, 2004). To overcome barrier function for enhanced permeation and delivery of these natural ingredients, many studies have investigated the use of nanotechnology, in particular the use of colloidal gold, for the green synthesis of gold nanoparticles (AuNPs). The surface of these nanoparticles is rapidly modified with the addition of chemical compounds or other bioactive molecules (Gupta and Rai, 2016). Gum arabic was used to maintain the electrostatic interactions and steric hinderance between the nanoparticles and the aqueous system in which they are dispersed, in order to prevent nanoparticle aggregation. The synthesis of nanoparticles using gum arabic also increases the biocompatibility, assists with reducing toxicity and is a cost-effective solution for prevention of nanoparticle aggregation (Chawla et al., 2020). In this study the antibacterial and anti-biofilm activity of nanoparticles coated with phytochemicals found in both an aqueous decoction of the powdered aerial plant material and methanolic extract of *H. odoratissimum* were investigated against the acne causing bacterium *C. acnes*. The study aimed to determine whether the AuNPs synthesized using *H. odoratissimum* would show similar antimicrobial and anti-biofilm activity when compared to that of the extract alone, which was previously reported by De Canha et al. (2020).

## Materials and Methods

### Chemicals and Reagents

The gold (III) salt (HAuCl_4_.3H_2_O), gum arabic (isolated from *Acacia seyal* and *Senegalia senegal*), sodium chloride (NaCl), cysteine, bovine serum albumin (BSA), tetracycline and 99.5% methanol were purchased from Sigma-Aldrich (Johannesburg, South Africa). The Dulbecco’s Modified Essential Medium and phosphate buffer saline (PBS) were purchased from Separations Scientific (Johannesburg, South Africa). The Brain Heart Infusion (BHI) agar and broth were purchased from Anatech Instruments (Pty) Ltd (Johannesburg, South Africa). The Oxoid AnaeroGen 2.5 L sachets were purchased from Thermofisher (Johannesburg, South Africa).

### Plant Extract Preparation and Gold Nanoparticle Synthesis

#### Plant Collection and Extraction

The aerial parts of *Helichrysum odoratissimum* (L.) Sweet were collected from Verulum (Kwazulu Natal, South Africa) in December. The species was identified by Ms. Magda Nel at the H.G.W.J. Schweickerdt Herbarium (University of Pretoria) and a herbarium voucher specimen was deposited (PRU 11863) in December 2012. The plant material was processed to a fine powder using an IKA MF 10 Basic grinder (4.0 mm sieve). The powdered plant material (3.5 kg) was macerated using 99.5% methanol (7 L) and agitated on a shaker for 48 h. The mixture was then filtered through a vacuum Büchner filter system and Whatman No. 1 filter paper. The methanolic extract was then concentrated under reduced pressure using a rotary evaporator (Büchi Rotavapor R-200). The dried methanolic extract was then logged into an extract library and stored at 4°C until further use.

#### Synthesis of Gold Nanoparticles (AuNPs)

The nanoparticles were synthesized according to the methods described by Khoobchandani et al. (2013), with minor modifications.

For complete green synthesis, 300 mg of dried powdered aerial parts of *Helichrysum odoratissimum* were added to a 50 mL glass beaker, with 18 mL of distilled water (dH_2_O). The mixture was then stirred and heated to 45°C and allowed to extract for 15 min. The plant material was then separated from the mixture using a using a vacuum Büchner filter system and Whatman No. 1 filter paper. The filtrate was placed back on the heating plate and maintained at 45°C, while stirring. For the stabilized gold nanoparticles, 36 mg of gum arabic was added to the filtrate and allowed to dissolve completely, after which 300 μL of a 0.1 M solution of HAuCl_4_.3H_2_O was added and allowed to react for a further 15 min. Successful synthesis of gold nanoparticles was observed by the formation of a deep purple color, symbolic of a red wine (HO Powder AuNPs + GA). The non-stabilized nanoparticles were synthesized using the same method, merely omitting the addition of the gum arabic (HO Powder AuNPs − GA).

For preparation of the gold nanoparticles from the methanolic extract, 1 mL (18 mg/mL stock) was added to 17 mL of distilled water (dH_2_O) in a 50 mL glass beaker. The mixture was then stirred and heated to 45°C. For the stabilized nanoparticles, 36 mg of gum arabic was added and allowed to dissolve completely, after which 300 μL of a 0.1 M HAuCl_4_.3H_2_O solution was added and allowed to react for a further 15 min. Successful synthesis of gold nanoparticles was observed by the formation of a deep purple color, symbolic of a red wine (HO Powder AuNPs + GA). The non-stabilized extract nanoparticles were synthesized using the same method, merely omitting the addition of the gum arabic (HO Powder AuNPs − GA).

#### Characterization of Synthesized Nanoparticles

##### Ultraviolet-visual (UV-Vis) Spectroscopy

The formation of the AuNPs was confirmed by performing a full spectral scan. In a 24-well plate, 1 mL of a diluted dispersion form of the respective AuNPs (1:9 in distilled water) was added to each well. The full spectral absorbance scan was performed using a BIO-TEK power-wave XS plate reader (A.D.P., Weltevreden Park, South Africa) from 450 to 800 nm.

##### Stability of Synthesized AuNPs

Stability of the synthesized nanoparticles was determined in various buffer solutions and cell culture media, which mimic physiological environments. Various ratios of AuNPs to buffer solutions and culture media were tested (80:120 μL; 40:160 μL and 20:180 μL). The buffer solutions were comprised of 5% NaCl, 0.5% cysteine, 0.5% bovine serum albumin, and Dulbecco’s Modified Eagle’s Medium (DMEM). The stability was investigated at Day 0, 1, 4, and 7 by measuring a full UV spectral scan, reading the absorbance from 450 to 800 nm using a BIO-TEK power-wave XS plate reader (A.D.P., Weltevreden Park, South Africa).

##### High Resolution Transmission Electron Microscopy (TEM) Analysis

The shape, size and dispersion of each of the synthesized nanoparticles was determined using TEM. The nanoparticles were loaded on a carbon-coated copper grid by the addition of 5 μL of the dispersion form and allowed to dry overnight under a fume hood. After 24 h, the TEM grids were loaded in a JEOL JEM-ARM200F double Cs-corrected Transmission Electron Microscope equipped with a large solid angle energy dispersive spectrometer (EDS) (Akishima, Tokyo, Japan) and images were captured.

##### Dynamic Light Scattering (DLS) Analysis

The particle size distribution was measured by diluting the dispersion form of freshly synthesized AuNPs (1:14) in dH_2_O. Diluted nanoparticles were then added to a clean quartz curvette and analyzed using the Horiba Dynamic Light Scattering Particle Analyzer LB-550 (Horiba Ltd., Japan). Each of the synthesized nanoparticles were analyzed by three separate reads to obtain an average particle size. A blank of dH_2_O was used to reduce background signal.

##### Fourier Transform Infrared Spectroscopy (FTIR) Analysis

Synthesized AuNPs were centrifuged at 4000 rpm for 10 min and washed with distilled water. Centrifuged nanoparticles were then freeze dried under vacuum at −50°C and 0.2 atm. The HO-MeOH extract was used as a blank for the HO-MeOH AuNPs and dried aerial parts of *Helichrysum odoratissimum* was used as a blank for the HO-Powder AuNPs. The analysis also compensated for background noise, by blanking with an empty read (no sample). The % transmittance was detected over an infrared range of 650–4000 nm.

##### X-ray Diffraction (XRD) Analysis

Freeze-dried AuNPs were loaded on sample mounting stages using glass cover slips. The crystal structure analysis of HO-MeOH AuNPs and HO-Powder AuNPs was determined using a PANalytical X’Pert PRO (PANalytical, Almelo, Netherlands) by irradiating AuNPs with monochromatized Cu K_α_ radiation (λ = 1.54 Å) between 30 and 90° (2θ) with a step size of 0.02°. The voltage and current were set to 45 kV and 40 mA, respectively.

##### Thermogravimetric Analysis (TGA)

The thermal decay of HO-MeOH and HO-Powder AuNPs was determined using a TG Q500 V20.13 build 39 (TA Instruments, Wilmington, DE, United States). Prepared freeze-dried AuNPs (10–20 mg) were loaded on platinum sample pans and heated from 40 to 900°C with 30°C/min ramp set-up.

##### Zeta (ζ) Potential

The Zeta-potential of the HO-MeOH and HO-Powder AuNPs was determined using the Zetasizer Nano ZS (Malvern, UK). The AuNPs were dispersed in deionized water (150 μL) and sonicated for 5 min. Zeta-potential was determined at 25°C using a reusable “dip” cell system. The set parameters included a count rate of 2.3 k, 100 zeta runs, a measurement position of 4.5 mm and an attenuator index of 11.

##### Quantification of Total Phenolic Content of the Synthesized Nanoparticles

To successfully compare whether the activity of the nanoparticles was better than those of the HO-MeOH alone [previously published in De Canha et al. (2020)] the total phenolic content of the synthesized nanoparticles was determined using the Folin Cioalteau technique as described by Basma et al. (2011) with modifications. The standard curves were prepared using the HO-MeOH (with and without gum arabic) and the aqueous decoction (with and without gum arabic) as described in section “Synthesis of gold nanoparticles (AuNPs)” to calculate the extract equivalents in the synthesized nanoparticles. Briefly, twelve (12) two-fold serial dilutions of each crude extract were prepared in a 96-well plate in dH_2_O (100: 100 μL). To each dilution, 50 μL of a 7.5% w/v Na_2_CO_3_ solution was added, followed by the addition of 50 μL of a 10% v/v solution of Folin Cioalteau reagent (1 in 10 mL dH_2_O). Plates were then incubated in a 45°C oven for 45 min. The absorbance was then measured at 765 nm using the PerkinElmer VICTOR Nivo^TM^ plate reader (Perkin Elmer, Midrand, South Africa). Quantification of the phenolics in the AuNPs was performed for the highest tested concentration which was 25% v/v in each of the bioassays (Table 1 – Section “Biological activity of the synthesized nanoparticles”). For each extract and synthesized nanoparticle type, blanks were included which comprised of everything except the 10% v/v Folin Cioalteau solution.

**TABLE 1:** Calculation of the total phenolic content of the synthesized AuNPs as extract equivalents for the conversion of concentration represented as % v/v to μg/mL.

**Synthesized gold nanoparticle**	**Highest concentration tested for biological activity (% v/v)**	**Corresponding extract equivalent (μg/mL) calculated for 25% v/v**	**Resulting concentration ranges for the antimicrobial and anti-biofilm assays (μg/mL)**
HO-MeOH − GA	25	76.36	0.60–76.36
HO-MeOH + GA	25	106.84	0.83–106.84
HO-Powder − GA	25	285.14	2.23–285.14
HO-Powder + GA	25	668.75	5.22–668.75

#### Biological Activity of the Synthesized Nanoparticles

##### Antibacterial Testing Against Cutibacterium acnes (ATCC 6919)

The antibacterial activity of the AuNPs was determined using a modified broth microdilution assay according to the methods described by Moreno-Álvarez et al. (2010) and Tsai et al. (2010). Briefly, a 72 h culture of *Cutibacterium acnes* was prepared in BHI broth and was adjusted to obtain approximately 1 × 10^8^ colony-forming units (CFU)/mL (OD_600_ = 0.1). In a 96 well plate, 100 μL of the dispersion form of the AuNPs was serially diluted, two-fold in BHI broth to obtain eight concentrations (0.20–25% v/v). The phenolic content was used to determine the correlating concentration ranges in μg/mL which are given in Table 1. To each test sample, 100 μL of the prepared *C. acnes* inoculum was added. The controls included a media control of BHI broth, a vehicle control of dH_2_O, and a bacterial control with *C. acnes* only. Each concentration was tested in triplicate. The plates were then incubated anaerobically (Oxoid AnaeroGen 2.5 L sachet in an Anaerocult Jar) at 37°C for 72 h. After incubation, 20 μL of PrestoBlue^®^ reagent was added to each test sample and plates were incubated at 37°C for an additional 1 h (Lall et al., 2013). The minimum inhibitory concentration (MIC) was determined by reading the fluorescence at an excitation/emission of 560 nm/590 nm using the PerkinElmer VICTOR Nivo^TM^ system (Perkin Elmer, Midrand, South Africa).

##### Anti-biofilm Activity Against Cutibacterium acnes (ATCC 6919)

The inhibition of adhesion of *Cutibacterium acnes* (ATCC 6919) performed using the methods described by Coenye et al. (2007). Actively growing cultures of *C. acnes* were inoculated in sterile 96-well plates by adding 100 μL of inoculum (OD_600_ = 0.1) in BHI to each well. Following bacterial plating, 100 μL of AuNPs were added to achieve concentrations as described in Table 1. Plates were then incubated anaerobically for 72 h. Following incubation, the BHI media was removed and plates were gently washed with 100 μL Phosphate Buffered Saline (PBS) three times. Adhered cells were then fixed by the addition of 100 μL of refrigerated (4°C) 99.5% methanol (MeOH) for 15 min. The MeOH was removed and plates were then allowed to air dry for 20 min. Quantification of adhered cells was then performed by adding 100 μL of a 0.5% crystal violet solution for 20 min. The plates were then gently rinsed with distilled water to remove excess crystal violet and allowed to air dry once more. The bound crystal violet was then dissolved using 160 μL of a 33% glacial acetic acid solution. The optical density was then measured at 590 nm using a BIO-TEK Power-Wave XS multi-well reader (A.D.P., Weltevreden Park, South Africa) for quantification of adhered bacterial cells.

The ability of the *Cutibacterium acnes*, strain ATCC 6919 to form a biofilm has been previously reported by Coenye et al. (2007). Bacterial cultures were plated as described above in the adhesion inhibition protocol. After the 72 h adhesion step, the BHI media was removed and the plates were washed three times with 100 μL of PBS, to remove planktonic cells. Following the wash step, 200 μL of fresh BHI was added and plates were then incubated for an additional 72 h to allow for the development of a mature biofilm. After this second incubation the supernatant was removed and replaced with 200 μL of test sample in BHI broth at the concentration ranges described in Table 1. After 24 h of treatment the total biofilm biomass was quantified using crystal violet as described above.

### Statistical Analysis

For statistical analysis of the prevention of *Cutibacterium acnes* attachment and *C. acnes* biofilm disruption the data was analyzed using ANOVA, with a Dunnett’s comparison where all columns were compared with the untreated *C. acnes* control. Significant data was interpreted as follows: ^∗^*p* < 0.05, ^∗∗^*p* < 0.01, and ^∗∗∗^*p* < 0.001.

### Results and Discussion

The methanolic extract and the aqueous decoction of the dried aerial parts of *Helichrysum odoratissimum* plant material both contained phytochemical constituents with the ability to reduce the Gold (III) chloride salt resulting in the biosynthesis of gold nanoparticles. The reductive power of the HO-MeOH and HO-Powder extracts were observed visually with the reaction of the gold salt and the extracts producing a ruby red color resembling red wine, characteristic of the formation of gold nanoparticles (Islam et al., 2015). Plant extracts contain numerous compounds including sugars, phenols, amines, ketones, aldehydes, carboxylic acids and proteins with bio-reducing ability to form nanoparticles. The HO-MeOH extract and aqueous decoction were likely formed by the interaction of these compounds to produce the AuNPs stabilized on the surface of the gold ions. The shape and size of nanoparticles is controlled by several parameters including the concentration of plant extract/material, the metal salt used, pH, temperature, and incubation time in the reaction mixture (Siddiqi and Husen, 2017). Differences in shape and size could potentially impact biological activity of the synthesized nanoparticles. Therefore, characterization of the formed nanoparticles was performed using various techniques to determine whether the addition of a stabilizing agent, gum arabic had an influence on shape, size, and biological activity.

#### Ultra-Violet Spectroscopy

**FIGURE 1:**
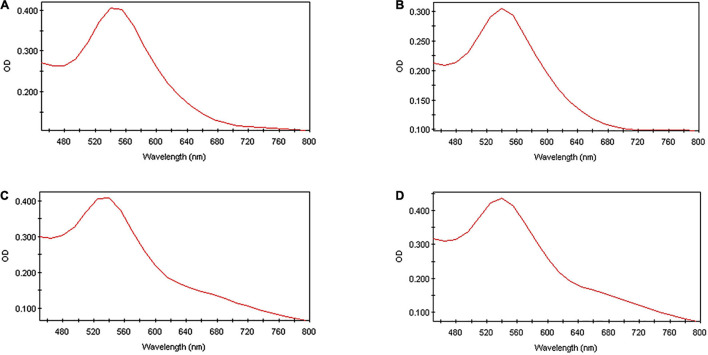
The ultraviolet spectra of **(A)** *Helichrysum odoratissimum* methanol extract + Gum arabic, **(B)** *H. odoratissimum* methanol extract – Gum arabic, **(C)** *H. odoratissimum* leaf powder + Gum arabic and **(D)** *H. odoratissimum* leaf powder – Gum arabic gold nanoparticles after synthesis (Day 0) from 450 to 800 nm.

The successful synthesis of gold nanoparticles was confirmed with a full spectral scan from 450 to 800 nm (Figure 1). The excitation of surface plasmon vibrations on the surface of the synthesized gold nanoparticles resulted in maximal peaks for all the AuNPs at 540 nm. The surface plasmon resonance of gold nanoparticles occurs within the visible light spectrum at approximately 520–540 nm and corresponds with the results observed for all the HO-MeOH and HO-Powder nanoparticles (Huang and El-Sayed, 2010; Siddiqi and Husen, 2017). There were no major differences in peak broadness or formation of peaks at longer wavelengths, indicating that there were low levels of nanoparticle aggregation. The peak maxima occurring at 540 nm correspond well with the formation of spherical nanoparticles, which was confirmed with TEM.

#### *In vitro* Stability Using Ultra-Violet Spectroscopy

**FIGURE 2:**
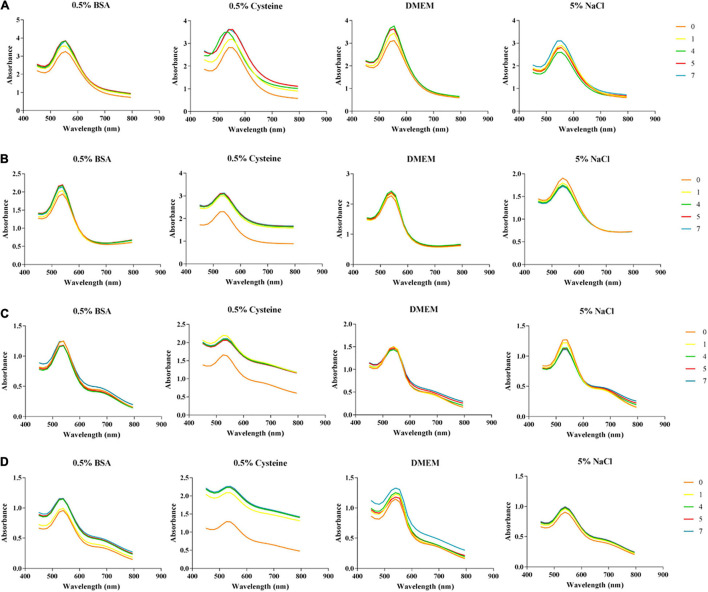
Effect of biological salts and additives on the stability of **(A)** *Helichrysum odoratissimum* methanol extract + Gum arabic, **(B)** *H. odoratissimum* methanol extract – Gum arabic, **(C)** *H. odoratissimum* leaf powder + Gum arabic and **(D)** *H. odoratissimum* leaf powder – Gum arabic gold nanoparticles at pH 7 from 450 to 800 nm.

The use of nanoparticles in biological applications, requires them to remain stable when exposed to certain biological environments. The use of salts and biological additives, mimicking these environments, are often used to provide valuable information about how these AuNPs could potentially behave in certain biological systems. Nanoparticles that are stable in these biological media do not agglomerate and will therefore exhibit minimal changes in their UV spectra. The changes in absorbance were recorded from synthesis Day (0) over a week, ending after Day (7) (Figure 2). The selected pH of the buffer solution was 7, to match the pH of the BHI media (pH 7.4 ± 0.2 at 25°C) used in the antibacterial and anti-biofilm biofilms. The overall trend for all the nanoparticles suggests that they were least stable in 0.5% cysteine solution. Upon comparison of the spectral changes between the two types of AuNPs, the HO-MeOH variations showed better stability with fewer changes in the surface plasmon resonance showing less shifts toward the red spectrum. The HO-Powder AuNPs, showed small tailing peaks in the red spectrum range which was indicative of increased nanoparticle size. These AuNPs also exhibited peak broadening or flattening which indicates increases in particle size (Elbagory et al., 2016). The AuNPs showed good stability in the 5% NaCl solution which suggests that the biological activity of the AuNPs will not be affected by the long incubation time required for antibacterial and anti-biofilm assays.

#### High Resolution Transmission Electron Microscopy (TEM) Analysis

**FIGURE 3:**
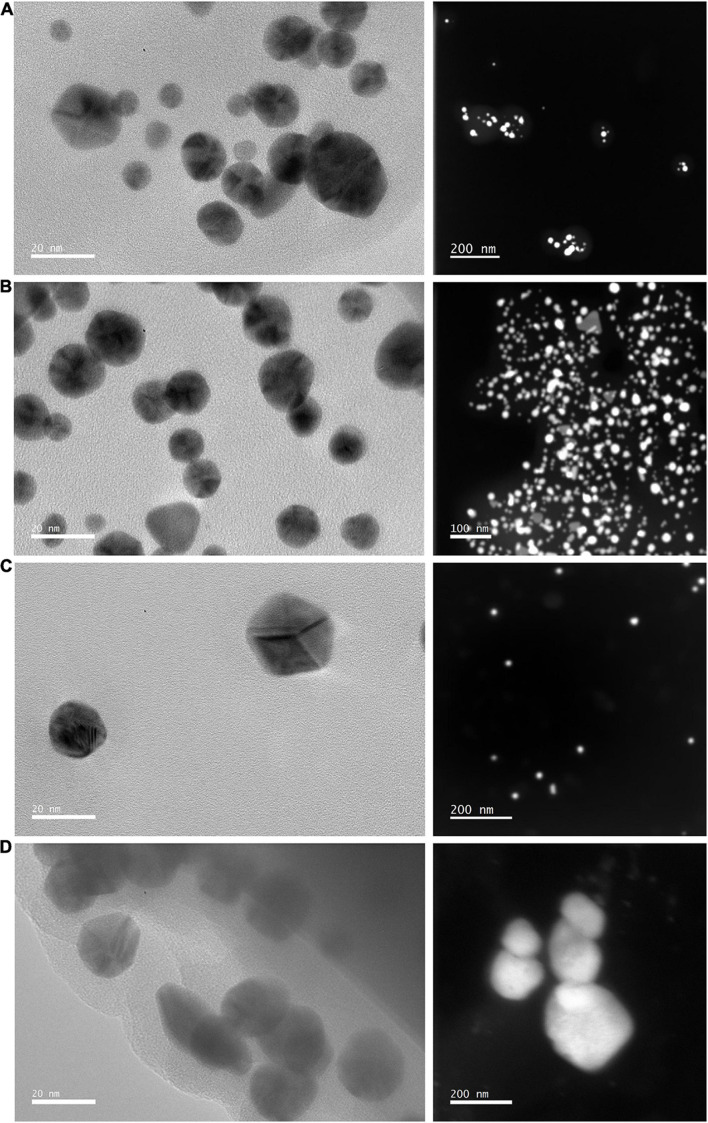
High Resolution Transmission electron micrographs and Scanning Transmission Electron micrographs (STEM) of **(A)** *Helichrysum odoratissimum* methanol extract + Gum arabic, **(B)** *H. odoratissimum* – Gum arabic gold nanoparticles, **(C)** *H. odoratissimum* leaf powder + Gum arabic, and **(D)** *H. odoratissimum* leaf powder – Gum arabic gold nanoparticles.

The transmission electron micrographs of the synthesized nanoparticles confirmed the formation of nanoparticles. Variations of shape and size were observed for both HO-MeOH and HO-Powder AuNPs, with spherical nanoparticles making up the majority for both types of AuNPs (Figures 3A,B). Variations in nanoparticle shape and size is common when using plant extracts as reducing agents and is often accompanied by observations where specific shapes are more dominant (Elbagory et al., 2016). In addition to the spherical nanoparticles, formation of triangular, hexagonal and some irregular shapes were observed for the HO-MeOH AuNPs (Figures 3A,B). The AuNPs formed using the dried aerial parts of *Helichrysum odoratissimum* were largely spherical, however, the formation of triangular, trapezoid and rod shapes were also observed (Figures 3C,D). The formation of nanoparticles at pH 8 are generally spherical or oval, considering distilled water was used in this study with a pH of approximately 7 this may help explain the shape of the formed nanoparticles. For AuNPs synthesized between 40 and 50°C not only spherical AuNPs, but also triangular, hexagonal and trapezoid AuNPs have been observed. Reaction times of 20–30 min have also been observed to form spherical, triangular, hexagonal, and trapezoid AuNPs (Siddiqi and Husen, 2017). Spherical shaped nanoparticles formed using the HO-MeOH + GA and HO-MeOH − GA (Figures 3A,B) were of similar size (∼20 nm). Upon comparison between the different types of nanoparticles it was observed that those formed using the fresh plant material were slightly smaller than those synthesized using the *H. odoratissimum* methanol extract. This was confirmed using DLS to determine particle hydrodynamic diameter and distribution.

#### Dynamic Light Scattering (DLS) Analysis

**FIGURE 4:**
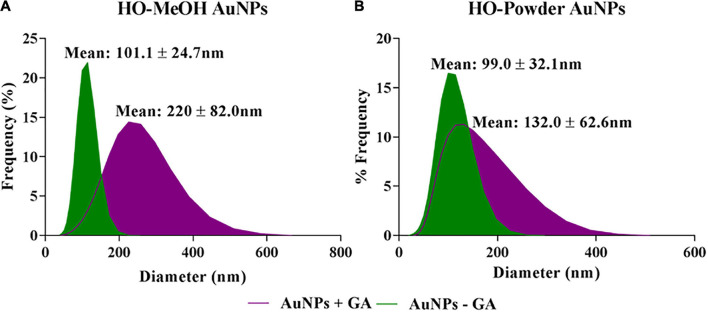
Hydrodynamic diameter of stabilized and un-stabilized **(A)** *Helichrysum odoratisimum* methanol extract and **(B)** *H. odoratissimum* leaf powder gold nanoparticles.

The dynamic light scattering technique was used to determine the hydrodynamic diameter of the nanoparticles. The HO-MeOH and HO-Powder with gum arabic (+GA) had mean hydrodynamic diameter sizes of 220.0 ± 82.0 nm and 132.0 ± 62.6 nm, respectively. The HO-MeOH and HO-Powder without gum arabic (−GA) were considerably smaller with mean hydrodynamic diameter sizes of 101.1 ± 24.7 nm and 99.0 ± 32.1 nm, respectively (Figure 4). This is most likely due to the ability of gum arabic to act as both a reducing agent and a stabilizing agent. The combined reducing ability of the plant phytochemicals with the stabilizing effect of the gum arabic would most likely result in the formation of nanoparticles with larger hydrodynamic diameters, indicating the presence of a stabilizing layer surrounding the AuNPs (Perde-Schrepler et al., 2016). There were no distinct differences in size or hydrodynamic diameter of the un-stabilized HO-MeOH and HO-Powder AuNPs (−GA) which also produced nanoparticles with large hydrodynamic diameters. The results correlate with that of a study by Nune et al. (2009) which showed that AuNPs synthesized using tea leaves showed a larger hydrodynamic diameter when stabilized with the addition of gum arabic.

#### Fourier Transform Infared Spectroscopy Analysis

**FIGURE 5:**
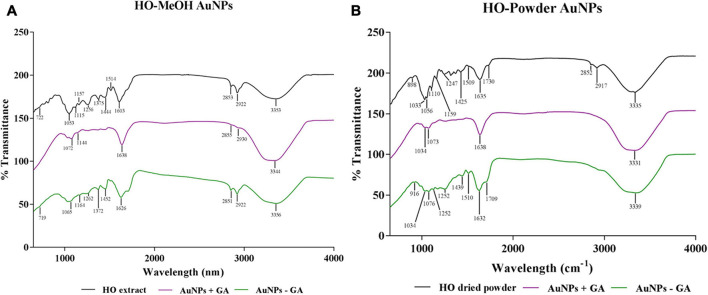
Fourier Transform Infrared spectra of *Helichrysum odoratisimum* methanol extract **(A)** and *H. odoratissimum* leaf powder **(B)** gold nanoparticles.

The FTIR spectra of the synthesized AuNPs provided possible mechanisms of nanoparticle formation, associated with specific functional groups. The spectra of the synthesized AuNPs were compared with the spectra of the methanolic extract and the dried plant material used for preparation of the decoction for the HO-MeOH and HO-Powder AuNPs, respectively. The shared peaks of the extract which are also present in the nanoparticles would indicate the functional groups of phytochemical constituents that were most likely to act as reducing and capping agents (Shankar et al., 2004). This technique provides information about the surface chemistry of the nanoparticles, through the detection of organic functional groups (Mittal et al., 2013). Compounds with specific functional groups, particularly those with the ability to form electrostatic or electrovalent bonds with anionic gold present in solution should be present on AuNP surfaces. There were peaks commonly shared between both HO-MeOH and HO-Powder AuNPs when compared to the methanol extract and dried *Helichrysum odoratissimum* plant material spectra, respectively (Figure 5). There were some differences in the stabilized (+GA) and un-stabilized (−GA) AuNPs. This is most likely due to the binding of gum arabic to the AuNP surface through efficient binding of hydrophilic arabinogalactan and hydrophobic glycoprotein components of this stabilizer, therefore, preventing the binding of some functional groups from the plant extract (Kattumuri et al., 2007). The HO-MeOH + GA AuNPs showed prominent peaks at 1034, 1073, 1638, and 3331 cm^–1^. This corresponded to the peaks in the methanol extract at 1033, 1056, 1635, and 3335 cm^–1^. The broadness and peak intensity of HO-MeOH + GA at 3331 cm^–1^ indicates the presence of O-H alcohol groups or phenol groups. The sharp and relatively low intensity of the peak at 1638 cm^–1^ relates to an alkene stretch (C = C). The peaks of 1072 and 1144 cm^–1^ are likely to correspond to the C-O ester stretch of carbonyl groups occurring from 1070 to 1150 cm^–1^. The HO-MeOH − GA AuNPs showed similar groups previously mentioned with the 3356 cm^–1^ peak indicating the presence of O-H stretching bonds of alcohol or phenol groups. The relatively strong peaks at 2851 and 2922 cm^–1^ correspond strongly with the C-H stretching bond alkane bond. The peak at 1626 cm^–1^ corresponds to the alkene stretching bond (C = C). The peak at 1065 cm^–1^ falls within the ester stretch of carbonyl groups (C-O).

A similar pattern was observed for the HO-Powder + GA and HO-Powder −GA AuNPs where the gum arabic played a role in the number of peaks. The most prominent peaks on FTIR spectra were the intense broad peaks of both+GA at 3331 cm^–1^ and −GA AuNPs at 3339 cm^–1^ corresponding to the peak at 3335 cm^–1^ in the powdered plant material indicating the presence of O-H stretching bond of alcohol or phenol groups. Both HO-Powder + GA and −GA showed peaks at 1638 cm^–1^ and 1632 cm^–1^, respectively. These correspond to the peak at 1635 cm^–1^ in the dried plant material which could indicate the presence of the alkene stretching bond (C = C). The bands at 1034 and 1073 cm^–1^ (HO-Powder + GA) and 1034 and 1076 cm^–1^ (HO-Powder −GA) correspond to the bands in the plant material at 1033 and 1056 cm^–1^ indicating the presence of an ester C-O stretch with the appearance of two bands in this range (Elia et al., 2014; Sathishkumar et al., 2016). Smitha et al. (2009) reported that the strong peak at 1037 cm^–1^ arises from C–O–C and C–OH vibrations, which suggests the interaction of plant proteins or enzymes playing a role in reduction of metal ions through their oxidative activity in the conversion of aldehydes into carboxylic acid.

#### X-ray Diffraction (XRD) Analysis

**FIGURE 6:**
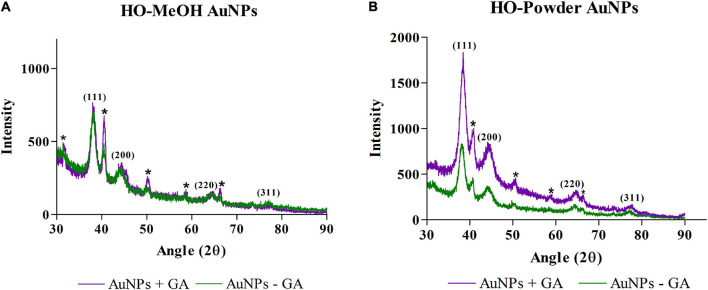
The X-Ray Diffraction chromatograms of *Helichrysum odoratissimum* methanol extract gold nanoparticles **(A)** and *H. odoratissimum* leaf powder **(B)** gold nanoparticles. * Represents unidentified peaks in the XRD spectra that do not match with the face-centred cubic crystal structure of gold.

X-Ray Diffraction (XRD) analysis was used to confirm the crystalline structure of AuNPs. The HO-MeOH and HO-Powder AuNPs showed similar XRD patterns. The diffraction angles for HO-MeOH AuNPs showed four main peaks at 38.2, 44.5, 64.7, and 77.2° and corresponded with the Bragg reflections (111), (200), (220), and (311) for the face centered cubic lattice structure of gold (Figure 6). The presence of gold was also confirmed using EDS analysis and the crystalline structure was confirmed by comparing the nanoparticle Scherrer ring patterns with that of gold (Supplementary Figures 1–4 and Figure 9). Similarly, the HO-Powder AuNPs exhibited diffraction angles at 38.5, 44.7, 64.9, and 77.7° (Figure 6). The XRD diffractogram was compared with that of gold (Au) as described by the International Centre for Diffraction Data (ICDD) from the JCPDS file (04-0784) (Gopalakrishnan and Raghu, 2014). These results showed that the synthesized gold nanoparticles were crystalline in nature. Similar diffraction angles were observed by Choi et al. (2014) with the use of catechin as a reducing agent. The unassigned peaks were similar to those observed in a study by Philip et al. (2011) which suggests that the crystallization of the extracts occurred at the surface of the nanoparticles. The sharpness and intensity of the (111) peak suggests that the orientation of the synthesized nanoparticles occurs predominantly in the (111) crystal lattice plane. Similar diffraction angles have been reported for AuNPs formed using cumin and gum arabic (Shalaby et al., 2015).

**FIGURE 7:**
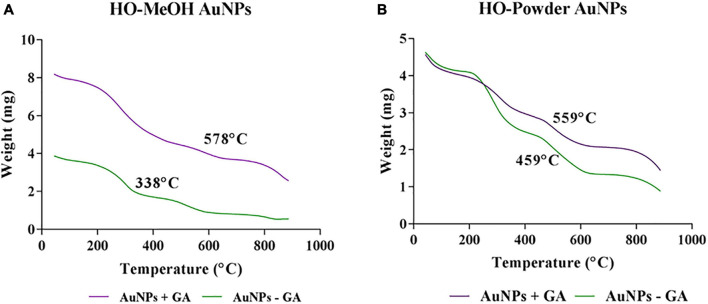
Thermal stability of **(A)** *Helichrysum odoratissimum* methanol extract gold nanoparticles and *H. odoratissimum* leaf powder **(B)** gold nanoparticles.

**FIGURE 8:**
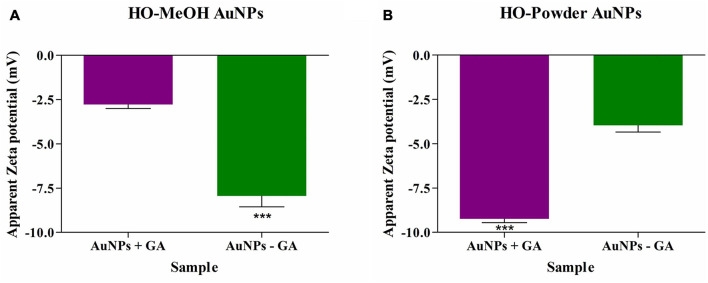
Zeta-potential of *Helichrysum odoratissimum* methanol extract **(A)** and *H. odoratissimum* leaf powder gold nanoparticles **(B)** gold nanoparticles. *** Is representative of the significance where *p*-value: *p* < 0.001.

**FIGURE 9:**
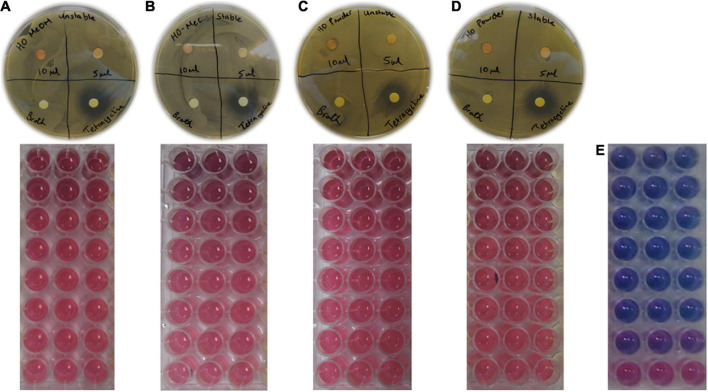
Disc diffusion and microdilution assay plates for **(A)** *Helichrysum odoratissimum* methanol extract AuNPs + Gum arabic, **(B)** *H. odoratissimum* methanol extract AuNPs – Gum arabic, **(C)** *H. odoratissimum* leaf powder AuNPs + Gum arabic, **(D)** *H. odoratissimum* leaf powder AuNPs – Gum arabic and **(E)** Tetracycline against *Cutibacterium acnes* grown on Brain Heart Infusion agar plates. Pink indicates *C. acnes* growth and purple/blue indicates bacterial inhibition.

#### Thermogravimetric Analysis

The thermal stability of the synthesized AuNPs was determined by monitoring the weight loss over time with increasing temperature. The initial decrease in weight loss occurring between 40 and 200°C can be attributed to the loss of absorbed water. The second most prominent decrease in weight is from 200 to 450°C, which is most likely due to the loss of organic compounds through combustion (Nadagouda et al., 2014). The temperatures shown on each graph indicate the temperature of which 50% of the initial weight is lost. In both the HO-MeOH + GA and HO-Powder + GA AuNPs these temperatures were significantly higher with 578 and 559°C when compared to those in the −GA variations which exhibited 50% weight loss at 338 and 459°C, respectively (Figure 7). The addition of gum arabic therefore plays a thermo-protective role for the nanoparticles. There were relatively low decreases in weight loss from 800 to 900°C which is most likely due to the remaining metallic gold residues (Mukundan et al., 2017).

#### Zeta (ζ) Potential

The zeta potential is not only indicative of the overall surface charge of metallic nanoparticles but is also an indicator of nanoparticle stability. Generally, highly negative or positive zeta-potential is indicative of high stability based on the repulsive forces between the nanoparticles, preventing agglomeration (Shabestarian et al., 2017). The HO-MeOH AuNPs with gum arabic (+GA) and without gum arabic (−GA) showed an overall negative potential of −2.78 mV and −7.93 mV, respectively. The HO-Powder AuNPs with gum arabic (+GA) and without gum arabic (−GA) showed similar results with overall negative surface charges of −9.23 mV and −3.96 mV, respectively (Figure 8 and Supplementary Figures 5–8). The HO-MeOH − GA showed better stability than the HO-Powder − GA potentially due to the extraction of more compounds using methanol when compared to the aqueous decoction. In the case of the HO-Powder AuNPs, the +GA nanoparticles were more stable due to the effective capping by the stabilizing gum arabic. The opposite was true for the HO-MeOH + GA nanoparticles. This is potentially due to the competitive binging of the compounds in the methanol extract creating stronger interactions with the Au^3+^ ions in comparison to the binding of polysaccharide gum arabic. The addition of gum arabic exhibited a positive effect on nanoparticle stability more so in the HO-Powder AuNPs. The addition of gum arabic to the nanoparticles synthesized using an aqueous decoction of *Helichrysum odoratissimum* leaf material showed a much more negative zeta-potential (−9.23 mV) in comparison with the HO-Powder − GA (with a charge of −3.96 mV). For the HO-MeOH AuNP variations the nanoparticles synthesized without gum arabic showed a more negative zeta-potential of −7.93 mV. This suggests that the compounds present in the methanol extract compete with gum arabic and show a higher reducing potential of the gold salt than that of gum arabic.

#### Quantification of Phenolic Content of Synthesized Nanoparticles

The phenolic content of each variation of the synthesized nanoparticles was determined as extract equivalents to convert the concentration of nanoparticles from % v/v to μg/mL, to compare whether the gold nanoparticles showed better activity. The calculated phenolic content was calculated for the highest tested concentration in the antibacterial and antibiofilm assays (25% v/v). Extract standard curves were prepared using the same amount of extract to dH_2_O and with or without gum arabic as described in section “Synthesis of gold nanoparticles (AuNPs)”. Each extract (with no gold salt added) was quantified for phenolic content by the addition of the Na_2_CO_3_ and 10% Folin Cioalteau reagents. The concentration and absorbance of each extract was plotted and a linear trendline was used to calculate the concentration of phenolics in the extracts. The phenolic content of the synthesized AuNPS were then also quantified by substituting the AuNP absorbance into the linear equation and calculating the concentration of extract present and then linked back to the phenolic content of the extract. The conversion of each concentration in % v/v to μg/mL is given in Table 1, for all the synthesized nanoparticles.

#### Biological Activity of Synthesized Nanoparticles

##### Antimicrobial Activity of Synthesized Nanoparticles Against Cutibacterium acnes

The use of AuNPs provides several advantages for antimicrobial activity as they are versatile in terms of size, shape, and functionality. Gold nanoparticles specifically, can be personalized to form specific sizes, altered to perform various biological functions while remaining biocompatible and can be traced intracellularly (Gupta et al., 2013). Considering these features, it was surprising to find that there are not many studies investigating the use of gold nanoparticles for their antibacterial activity against *Cutibacterium acnes*. There are, however, many studies reporting the broad-spectrum antimicrobial activity of gold and silver nanoparticles against human and animal pathogens (Mittal et al., 2013). The use of silver nanoparticles as antimicrobial agents, however, largely outweighs their gold counterparts. This is evident in the number of medical and commercial products made using silver. These products include silver nanoparticle powders, dressings, and even coated medical devices (Rai et al., 2009). Recent studies, however, have shown the potential of silver nanoparticles (at sub-lethal concentrations) to induce antibiotic resistance of *Staphylococcus aureus* and *Escherichia coli* toward ampicillin and Penicillin-Streptomycin. This, in addition to the environmental implications of silver (Kaweeteerawat et al., 2017) suggests that gold nanoparticles may be more beneficial. The use of some silver products has also shown to induce toxicity against keratinocytes and skin fibroblasts, an undesired effect, particularly when many treatment options for acne vulgaris rely largely on topical application of effective therapy (Burd et al., 2007). Due to the lack of information available for the use of AuNPs in treating acne, the antimicrobial activity of the synthesized HO-MeOH and HO-Powder AuNPs were determined against *C. acnes* (ATCC 6919). The dispersion form of the two synthesized AuNP variations showed no antimicrobial activity in the disc diffusion assay or the microdilution assay when tested against this skin pathogen. The positive control tetracycline was included to ensure that the assay was performed under sterile conditions. Tetracycline showed a considerably larger zone of inhibition when compared to the HO-MeOH and HO-Powder AuNPs (Figure 9). Gram-positive bacteria have been reported to have an increased resistance to the antimicrobial mechanisms of nanoparticles. This resistance is attributed to the thickness of the peptidoglycan layer present in Gram-positive microorganism cell walls. The cell walls of Gram-positive and Gram-negative bacteria are negatively charged. The overall negative zeta-potential of the synthesized nanoparticles indicates an overall negative surface charge of the AuNPs, suggesting that they will not fuse with bacterial cell walls, a mechanism which is necessary for damage (Slavin et al., 2017). A study by Gao et al. (2014) reported that AuNPs with cationic (+) surface charges show better antimicrobial activity. Cationic liposomes stabilized with anionic gold nanoparticles fuse with bacteria at acidic pH, which can be highly effective as a topical delivery system for *C. acnes*, highlighting the importance of nanoparticle surface charge and antimicrobial activity. Although the synthesized AuNPs did not show antimicrobial activity, these nanoscale particles have shown activity against bacterial biofilms and warranted the investigation of the inhibition of *C. acnes* biofilm formation and disruption of mature biofilms (Baptista et al., 2018).

##### Anti-biofilm Activity of Synthesized Nanoparticles Against Cutibacterium acnes

**FIGURE 10:**
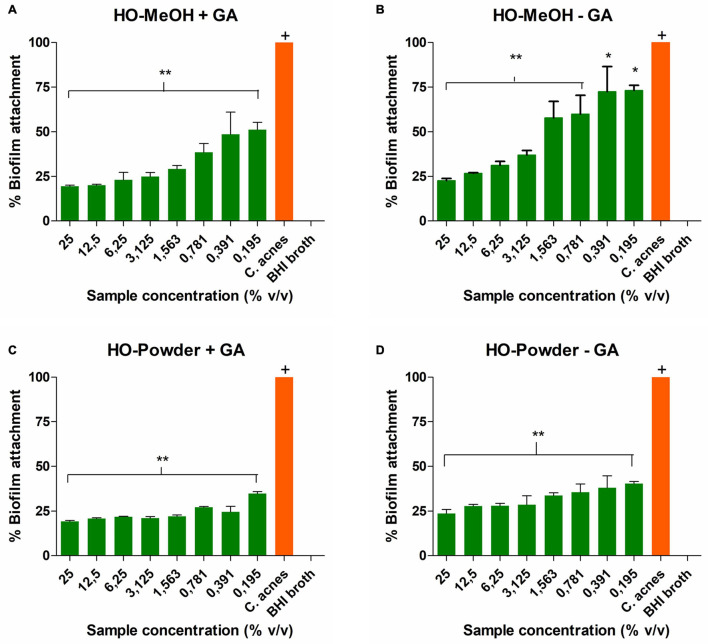
The percentage adhesion of *Cutibacterium acnes* treated for 72 h with **(A)** *Helichrysum odoratissimum* methanol extract AuNPs + Gum arabic, **(B)** *H. odoratissimum* methanol extract AuNPs – Gum arabic, **(C)** *H. odoratissimum* leaf powder AuNPs + Gum arabic, **(D)** *H. odoratissimum* leaf powder AuNPs – Gum arabic compared to the untreated *C. acnes* control. * Represents significance where *p* < 0.05, ** represents significance where *p* < 0.01, ^+^ represents the control to which all other samples were compared.

The HO-MeOH AuNPs showed better inhibition against *Cutibacterium acnes* cell adhesion when compared to HO-Powder AuNPs. The inhibition of initial cell adherence prevents the formation of biofilm. The minimum biofilm inhibitory concentration (MBIC_50_) was defined as the concentration required to inhibit 50% of biofilm formation. The MBIC_50_ for HO-MeOH − GA and HO-MeOH + GA were 1.79 ± 0.78% v/v and 0.22 ± 0.16% v/v, respectively (Figure 10). The anti-adhesion activity of the HO-MeOH + GA AuNPs was significantly better than the HO-MeOH − GA variation. The activity of HO-MeOH against the prevention of biofilm formation by targeting bacterial cell adhesion was demonstrated in a previous study (De Canha et al., 2020). It is possible the phytochemical constituents responsible for the anti-adhesion activity, are also those responsible for the reduction of the gold salt and the formation and stabilization of the nanoparticles. This could explain their ability to inhibit biofilm formation by preventing microbial adhesion to the surface. In contrast to the HO-MeOH only extract, this activity is most likely due to the effective binding of the compounds to the gold surface and not the antimicrobial activity against *C. acnes*. The presence of teichoic and teichuronic acids in the peptidoglycan layer of Gram-positive bacteria suggests that the overall surface charge of *C. acnes* biofilm would be anionic (negative) (Martin et al., 2015). This could explain why the HO-MeOH + GA AuNPs exhibited better anti-adhesion activity. The presence of larger negatively charged HO-MeOH + GA AuNPs increases electrostatic repulsion between the polystyrene surface and *C. acnes* cells as the nanoparticles may settle at the bottom of the plate (Rzhepishevska et al., 2013). The HO-Powder AuNPs showed more effective inhibition of *C. acnes* cell adhesion to the polystyrene surface. This could be explained by their strongly negative zeta-potentials which were higher than the HO-MeOH AuNPs variations. The combination of strong negative zeta-potential and slightly larger HO-Powder + GA AuNPs was most likely responsible for the anti-adhesion properties of these nanoparticles.

**FIGURE 11:**
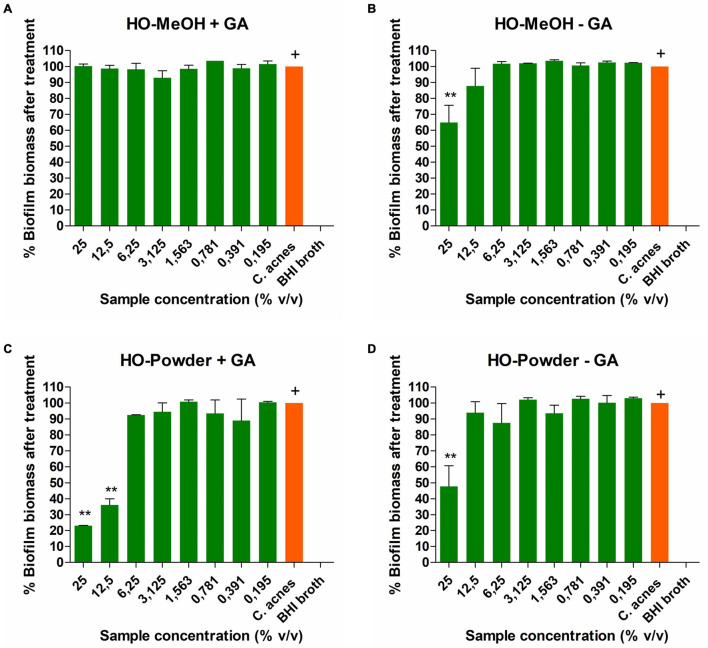
Dose-dependent disruption of mature *Cutibacterium acnes* biofilm after 72 h by **(A)** *Helichrysum odoratissimum* methanol extract AuNPs + Gum arabic, **(B)** *H. odoratissimum* methanol extract AuNPs – Gum arabic, **(C)** *H. odoratissimum* leaf powder AuNPs + Gum arabic, **(D)** *H. odoratissimum* leaf powder AuNPs – Gum arabic compared to the untreated *C. acnes* control. ** Represents significance where *p* < 0.01, ^+^ represents the control to which all other samples were compared.

The disruption of *Cutibacterium acnes* biofilms showed dose-dependent response for all the nanoparticles (Figure 11). The HO-MeOH − GA AuNPs showed better activity on biofilm biomass reduction when compared to the HO-MEOH + GA. This could be due to the smaller size of the HO-MeOH − GA which enabled them to interact with the biofilm extracellular matrix, causing slight reductions in the biofilm. The IC_50_ was 22.01 ± 6.13% v/v for HO-MeOH − GA, while the HO-MeOH + GA did not inhibit the biofilm biomass effectively, even at the highest concentration of 25% v/v. The HO-Powder + GA exhibited an IC_50_ of 11.78 ± 1.78% v/v, whereas HO-Powder − GA showed no biofilm disruption at the highest concentration. Previous reports on the anti-biofilm activity of GA capped silver nanoparticles, showed inhibition against the *Pseudomonas aeruginosa* biofilms (Jaiswal et al., 2015).

The results confirm that observed in the study by De Canha et al. (2020) where even the nanoparticles show better inhibition of *Cutibacterium acnes* adhesion to the 96-well plate rather than disruption of the mature biofilms. The phenolic content of the highest tested concentration (25% v/v) was used to convert the minimum biofilm inhibitory concentration (MBIC_50_) from % v/v to μg/mL of extract equivalents. One can see in Table 2, that the adhesion of *C. acnes* is inhibited by both HO-MeOH and HO-Powder AuNPs. The HO-MeOH + GA showed the highest anti-adhesion properties with an MBIC_50_ of 0.05 μg/mL. The biofilm disruption MBIC_50_ for the HO-MeOH − GA and HO-Powder + GA AuNPs was well above the MIC for the methanolic extract alone indicating that the bound constituents on the AuNPs are not those responsible for the antimicrobial activity of the extract in its crude form. This would be particularly useful to combat the resistance associated with biocidal or biostatic agents for the treatment of microbial infections, since the AuNPs have no antimicrobial activity and are still able to inhibit both attachment of *C. acnes in vitro* and disrupt the biofilm structure.

**TABLE 2:** Antibiofilm activity of synthesized gold nanoparticles against *Cutibacterium acnes* adhesion and biofilm disruption.

**Synthesized gold nanoparticles**	**MBIC_50_ in% v/v**	**MBIC_50_ μg/mL based on phenolic content of each nanoparticle compared with respective extract used in synthesis**
**Inhibition of *C. acnes* adhesion**		
HO-MeOH − GA	1.79	0.59
HO-MeOH + GA	0.22	0.05
HO-Powder − GA	<0.20	<2.23
HO-Powder + GA	<0.20	<5.22
***C. acnes* biofilm disruption**		
HO-MeOH − GA	22.01	67.23
HO-MeOH + GA	No inhibition	No inhibition
HO-Powder − GA	No inhibition	No inhibition
HO-Powder + GA	11.78	315.12
		

## Conclusion

The synthesis of gold nanoparticles using both the dried plant material and the methanol extract of *Helichrysum odoratissimum* resulted in the reduction of the gold salt. The use of *H. odoratissimum* in the nanoparticle form exhibited similar anti-biofilm activity when compared to the biological activity of the methanol extract as previously reported by De Canha et al. (2020). The formation of nanoparticles with plant extracts or dried plant material result in several shapes, sizes and therefore explained the varied functionality. The use of the HO-MeOH and HO-Powder AuNPs showed better efficacy against preventing biofilm formation through reduced *Cutibacterium acnes* adhesion as opposed to eradicating mature biofilms. The study provides a platform for future work to optimize the synthetic process, which is dependent on several parameters including pH, temperature, run time of reduction reaction, concentration of plant extract, and concentration of gold salt. The combinations and number of phytochemicals able to bind to the surface of gold warrants, further investigation of individual components of *H. odoratissimum* which could help elucidate exact mechanism related activity. This study is the first report of the use of the South African species, *H. odoratissimum*, in the synthesis of nanoparticles, which is surprising since the Helichrysum genus contains many species with potentially unexplored applications for cosmetics. The study emphasizes the ability of *H. odoratissimum* AuNPs for the prevention of *C. acnes* cell adhesion which is the preliminary step in biofilm formation. Future considerations will include the determination of the mechanism of action of the AuNPs against biofilm attachment by determining the effects of AuNP treatment on the extracellular matrix components of *C. acnes* biofilms and morphological changes to the biofilm using Scanning Electron Microscopy prior to attachment.

## Data Availability Statement

The raw data supporting the conclusions of this article will be made available by the authors, without undue reservation.

## Author Contributions

MD synthesized the nanoparticles with the help of VT and KK, conducted the biological testing, and compiled the manuscript with the help and supervision of NL. VM, MK, SR, and RR ran the nanoparticle characterization experiments with MD. AJ ran the HRTEM and EDS analysis of the synthesized nanoparticles. All authors contributed to the article and approved the submitted version.

## Conflict of Interest

The authors declare that the research was conducted in the absence of any commercial or financial relationships that could be construed as a potential conflict of interest.

## Publisher’s Note

All claims expressed in this article are solely those of the authors and do not necessarily represent those of their affiliated organizations, or those of the publisher, the editors and the reviewers. Any product that may be evaluated in this article, or claim that may be made by its manufacturer, is not guaranteed or endorsed by the publisher.

## References

[B1] Baptista P. V. McCusker M. P. Carvalho A. Ferreira D. A. Mohan N. M. Martins M. (2018). Nano-strategies to fight multidrug resistant bacteria-A battle of the titans. *Front. Microbiol.* 9:1441. 10.3389/fmicb.2018.01441 30013539 PMC6036605

[B2] Basma A. A. Zakaria Z. Latha L. Y. Sasidharan S. (2011). Antioxidant activity and phytochemical screening of the methanol extracts of Euphorbia hirta L. *Asian Pac. J. Trop. Med.* 4 386–390. 10.1016/s1995-7645(11)60109-0 21771682

[B3] Burd A. Kwok C. H. Hung S. C. Chan H. S. Gu H. Lam W. K. (2007). A comparative study of the cytotoxicity of silver-based dressings in monolayer cell, tissue explant, and animal models. *Wound Repair Regen.* 15 94–104. 10.1111/j.1524-475x.2006.00190.x 17244325

[B4] Byrd A. L. Belkaid Y. Segre J. A. (2018). The human skin microbiome. *Nat. Rev. Microbiol.* 16 143–155. 29332945 10.1038/nrmicro.2017.157

[B5] Chawla P. Kumar N. Bains A. Dhull S. B. Kumar M. Kaushik R. (2020). Gum arabic capped copper nanoparticles: synthesis, characterization, and applications. *Int. J. Biol. Macromol.* 146 232–242. 10.1016/j.ijbiomac.2019.12.260 31904465

[B6] Choi Y. Choi M. J. Cha S. H. Kim Y. S. Cho S. Park Y. (2014). Catechin-capped gold nanoparticles: green synthesis, characterization, and catalytic activity toward 4-nitrophenol reduction. *Nanoscale Res. Lett.* 9 103–110. 10.1186/1556-276x-9-103 24589224 PMC3944744

[B7] Coenye T. Peeters E. Nelis H. J. (2007). Biofilm formation by Propionibacterium acnes is associated with increased resistance to antimicrobial agents and increased production of putative virulence factors. *Res. Microbiol.* 158, 386–392. 10.1016/j.resmic.2007.02.001 17399956

[B8] De Canha M. N. Komarnytsky S. Langhansova L. Lall N. (2020). Exploring the anti-acne potential of impepho [Helichrysum odoratissimum (L.) Sweet] to combat cutibacterium acnes virulence. *Front. Pharmacol.* 10:1559. 10.3389/fphar.2019.01559 32082144 PMC7002546

[B9] De Wet H. Nciki S. van Vuuren S. F. (2013). Medicinal plants used for the treatment of various skin disorders by a rural community in northern Maputaland, South Africa. *J. Ethnobiol. Ethnomed.* 9:51. 10.1186/1746-4269-9-51 23870616 PMC3724715

[B10] Elbagory A. M. Cupido C. N. Meyer M. Hussein A. A. (2016). Large scale screening of Southern African plant extracts for the green synthesis of gold nanoparticles using microtitre-plate method. *Molecules* 21:1498. 10.3390/molecules21111498 27834835 PMC6273790

[B11] Elia P. Zach R. Hazan S. Kolusheva S. Porat Z. E. Zeiri Y. (2014). Green synthesis of gold nanoparticles using plant extracts as reducing agents. *Int. J. Nanomed.* 9 4007–4021. 10.2147/ijn.s57343 25187704 PMC4149460

[B12] Gao W. Vecchio D. Li J. Zhu J. Zhang Q. Fu V. (2014). Hydrogel containing nanoparticle-stabilized liposomes for topical antimicrobial delivery. *ACS Nano* 8 2900–2907. 10.1021/nn500110a 24483239 PMC4004330

[B13] Gopalakrishnan R. Raghu K. (2014). Biosynthesis and characterization of gold and silver nanoparticles using milk thistle (Silybum marianum) seed extract. *J. Nanosci.* 2014 1–8. 10.1155/2014/905404

[B14] Gupta R. Rai B. (2016). Penetration of gold nanoparticles through human skin: unraveling its mechanisms at the molecular scale. *J. Phys. Chem. B* 120 7133–7142. 10.1021/acs.jpcb.6b03212 27362257

[B15] Gupta S. Bansal R. Gupta S. Jindal N. Jindal A. (2013). Nanocarriers and nanoparticles for skin care and dermatological treatments. *Indian Dermatol. Online J.* 4:267. 10.4103/2229-5178.120635 24350003 PMC3853888

[B16] Huang X. El-Sayed M. A. (2010). Gold nanoparticles: optical properties and implementations in cancer diagnosis and photothermal therapy. *J. Adv. Res.* 1 13–28. 10.1016/j.jare.2010.02.002

[B17] Islam N. U. Jalil K. Shahid M. Rauf A. Muhammad N. Khan A. (2015). Green synthesis and biological activities of gold nanoparticles functionalized with Salix alba. *Arab. J. Chem.* 12 2914–2925. 10.1016/j.arabjc.2015.06.025

[B18] Jaiswal S. Bhattacharya K. McHale P. Duffy B. (2015). Dual effects of β-cyclodextrin-stabilised silver nanoparticles: enhanced biofilm inhibition and reduced cytotoxicity. *J. Mater. Sci. Mater. Med.* 26, 52–61. 10.1007/s10856-014-5367-1 25596861

[B19] Kattumuri V. Katti K. Bhaskaran S. Boote E. J. Casteel S. W. Fent G. M. (2007). Gum arabic as a phytochemical construct for the stabilization of gold nanoparticles: in vivo pharmacokinetics and X-ray-contrast-imaging studies. *Small* 3 333–341. 10.1002/smll.200600427 17262759

[B20] Kaweeteerawat C. Na Ubol P. Sangmuang S. Aueviriyavit S. Maniratanachote R. (2017). Mechanisms of antibiotic resistance in bacteria mediated by silver nanoparticles. *J. Toxicol. Environ. Health A* 80 1276–1289. 10.1080/15287394.2017.1376727 29020531

[B21] Khoobchandani M. Zambre A. Katti K. Lin C. H. Katti K. V. (2013). Green nanotechnology from brassicaceae: development of broccoli phytochemicals–encapsulated gold nanoparticles and their applications in nanomedicine. *Int. J. Green Nanotechnol.* 1, 1–15. 10.1177/1943089213509474

[B22] Lall N. Heneley-Smith C. J. de Canha M. N. Oosthuizen C. B. Berrington D. (2013). Viability reagent, prestoblue, in comparison with other available reagents, utilized in cytotoxicity and antimicrobial assays. *Int. J. Microbiol.* 2013 1–5. 10.1155/2013/420601 23653650 PMC3638707

[B23] Lourens A. C. U. Reddy D. Başer K. H. C. Viljoen A. M. Van Vuuren S. F. (2004). In vitro biological activity and essential oil composition of four indigenous South African Helichrysum species. *J. Ethnopharmacol.* 95 253–258. 10.1016/j.jep.2004.07.027 15507345

[B24] Martin C. LiLow W. Gupta A. Cairul Iqbal Mohd Amin M. Radecka I Britland S. T. (2015). Strategies for antimicrobial drug delivery to biofilm. *Curr. Pharm. Des.* 21 43–66. 10.2174/1381612820666140905123529 25189862

[B25] McDowell A. Nagy I. Magyari M. Barnard E. Patrick S. (2013). The opportunistic pathogen Cutibacterium acnes: insights into typing, human disease, clonal diversification and CAMP factor evolution. *PLoS One* 8:e70897. 10.1371/journal.pone.0070897 24058439 PMC3772855

[B26] Mittal A. K. Chisti Y. Banerjee U. C. (2013). Synthesis of metallic nanoparticles using plant extracts. *Biotechnol. Adv.* 31 346–356. 10.1016/j.biotechadv.2013.01.003 23318667

[B27] Moreno-Álvarez S. A. Martínez-Castañón G. A. Niño-Martínez N. Reyes-Macías J. F. Patiño-Marín N. Loyola-Rodríguez J. P. (2010). Preparation and bactericide activity of gallic acid stabilized gold nanoparticles. *J. Nanopart. Res.* 12 2741–2746. 10.1007/s11051-010-0060-x

[B28] Mukundan D. Mohankumar R. Vasanthakumari R. (2017). Comparative study of synthesized silver and gold nanoparticles using leaves extract of Bauhinia tomentosa Linn and their anticancer efficacy. *Bull. Mater. Sci.* 40 335–344. 10.1007/s12034-017-1376-2

[B29] Nadagouda M. N. Iyanna N. Lalley J. Han C. Dionysiou D. D. Varma R. S. (2014). Synthesis of silver and gold nanoparticles using antioxidants from blackberry, blueberry, pomegranate, and turmeric extracts. *ACS Sustain. Chem. Eng.* 2 1717–1723. 10.1021/sc500237k

[B30] Nune S. K. Chanda N. Shukla R. Katti K. Kulkarni R. R. Thilakavathy S. (2009). Green nanotechnology from tea: phytochemicals in tea as building blocks for production of biocompatible gold nanoparticles. *J. Mater. Chem.* 19 2912–2920. 10.1039/b822015h 20161162 PMC2737515

[B31] Perde-Schrepler M. David L. Olenic L. Potara M. Fischer-Fodor E. Virag P. (2016). Gold nanoparticles synthesized with a polyphenols-rich extract from cornelian cherry (Cornus mas) fruits: effects on human skin cells. *J. Nanomater.* 2016 1–13. 10.1155/2016/6986370

[B32] Philip D. Unni C. Aromal S. A. Vidhu V. K. (2011). Murraya koenigii leaf-assisted rapid green synthesis of silver and gold nanoparticles. *Spectrochim. Acta A Mol. Biomol. Spectrosc.* 78 899–904. 10.1016/j.saa.2010.12.060 21215687

[B33] Rai M. Yadav A. Gade A. (2009). Silver nanoparticles as a new generation of antimicrobials. *Biotechnol. Adv.* 27 76–83. 10.1016/j.biotechadv.2008.09.002 18854209

[B34] Ribeiro A. S. Estanqueiro M. Oliveira M. B. Sousa Lobo J. M. (2015). Main benefits and applicability of plant extracts in skin care products. *Cosmetics* 2 48–65. 10.3390/cosmetics2020048

[B35] Rzhepishevska O. Hakobyan S. Ruhal R. Gautrot J. Barbero D. Ramstedt M. (2013). The surface charge of anti-bacterial coatings alters motility and biofilm architecture. *Biomater. Sci.* 1 589–602. 10.1039/c3bm00197k 32481834

[B36] Sahdo B. Särndahl E. Elgh F. Söderquist B. (2013). Cutibacterium acnes activates caspase-1 in human neutrophils. *APMIS* 121 652–663. 23278288 10.1111/apm.12035

[B37] Sathishkumar G. Jha P. K. Vignesh V. Rajkuberan C. Jeyaraj M. Selvakumar M. (2016). Cannonball fruit (Couroupita guianensis, Aubl.) extract mediated synthesis of gold nanoparticles and evaluation of its antioxidant activity. *J. Mol. Liq.* 215 229–236. 10.1016/j.molliq.2015.12.043

[B38] Shabestarian H. Homayouni-Tabrizi M. Soltani M. Namvar F. Azizi S. Mohamad R. (2017). Green synthesis of gold nanoparticles using Sumac aqueous extract and their antioxidant activity. *Mater. Res.* 20 264–270. 10.1590/1980-5373-mr-2015-0694

[B39] Shalaby T. I. El-Dine R. S. S. El-Gaber S. A. A. (2015). Green synthesis of gold nanoparticles using cumin seeds and gum arabic: studying their photothermal efficiency. *Nanosci. Nanotechnol.* 5 89–96.

[B40] Shankar S. S. Rai A. Ahmad A. Sastry M. (2004). Rapid synthesis of Au, Ag, and bimetallic Au core–Ag shell nanoparticles using Neem (Azadirachta indica) leaf broth. *J. Colloid Interface Sci.* 275 496–502. 10.1016/j.jcis.2004.03.003 15178278

[B41] Siddiqi K. S. Husen A. (2017). Plant response to engineered metal oxide nanoparticles. *Nanoscale Res. Lett.* 12, 1–18. 10.1186/s11671-017-1861-y 28168616 PMC5293712

[B42] Slavin Y. N. Asnis J. Häfeli U. O. Bach H. (2017). Metal nanoparticles: understanding the mechanisms behind antibacterial activity. *J. Nanobiotechnol.* 15 65–85. 10.1186/s12951-017-0308-z PMC5627441 28974225

[B43] Smitha S. L. Philip D. Gopchandran K. G. (2009). Green synthesis of gold nanoparticles using Cinnamomum zeylanicum leaf broth. *Spectrochim. Acta A Mol. Biomol. Spectrosc.* 74 735–739. 10.1016/j.saa.2009.08.007 19744880

[B44] Swelankomo N. (2004). *Helichrysum odoratissimum (L.) Sweet* [Online]. Available online at: http://pza.sanbi.org/helichrysum-odoratissimum (accessed July 17, 2018).

[B45] Tsai T. H. Tsai T. H. Wu W. H. Tseng J. T. P. Tsai P. J. (2010). In vitro antimicrobial and anti-inflammatory effects of herbs against Cutibacterium acnes. *Food Chem.* 119 964–968. 10.1016/j.foodchem.2009.07.062

[B46] Tuchayi S. M. Makrantonaki E. Ganceviciene R. Dessinioti C. Feldman S. R. Zouboulis C. C. (2015). Acne vulgaris. *Nat. Rev. Dis. Primers* 1:15029. 27189872 10.1038/nrdp.2015.29

