# Supplementary material for: The Activity of Gold Nanoparticles Synthesized Using Helichrysum odoratissimum Against Cutibacterium acnes Biofilms
Source: Front Cell Dev Biol. 2021 Sep 13;9:675064. doi: 10.3389/fcell.2021.675064 (PMC8473823; doi:10.3389/fcell.2021.675064)
Supplement: Supplementary file 1 [file Data_Sheet_1.PDF]

Supplementary Data:

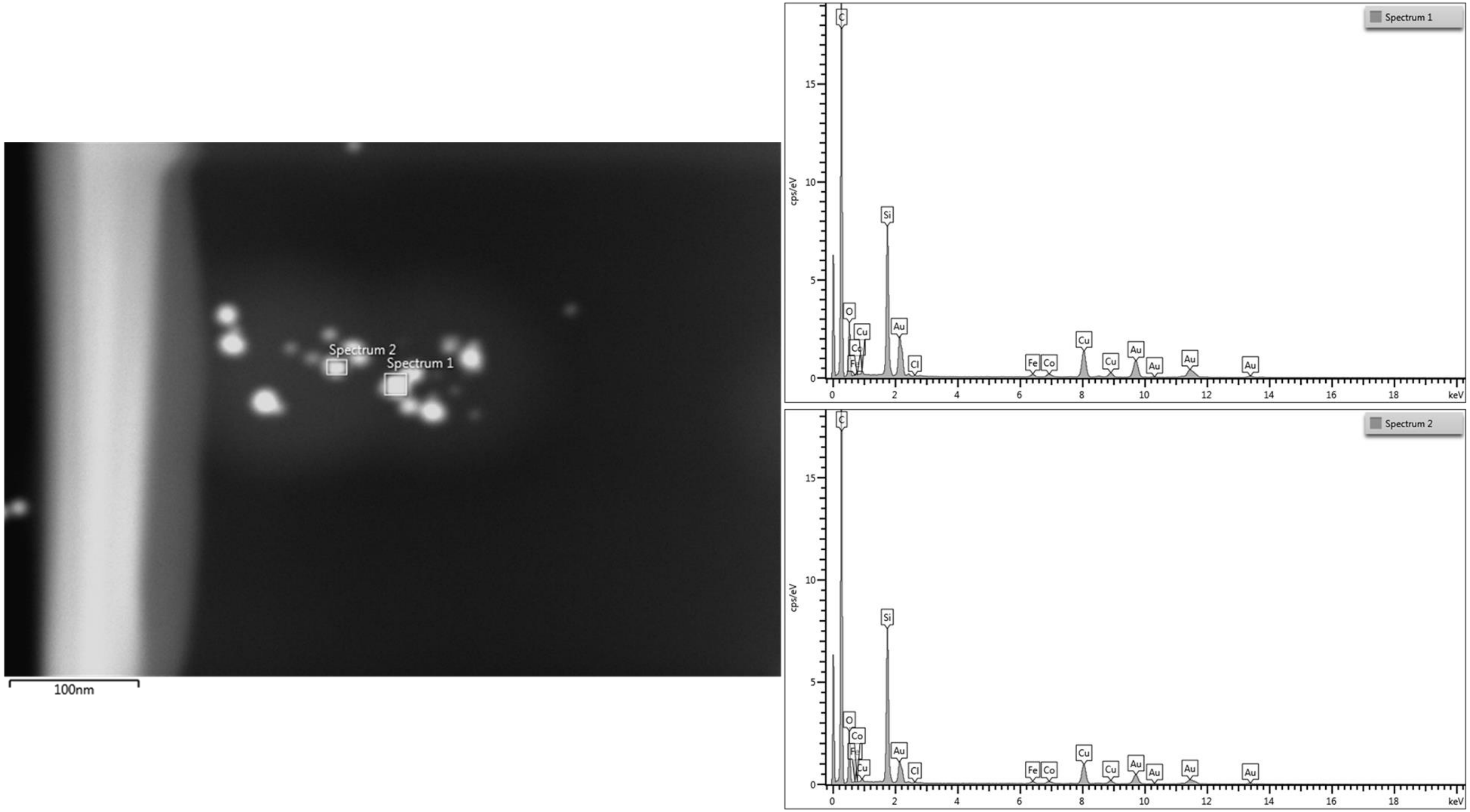

Figure 1: EDS spectra of HO-MeOH + GA AuNPs

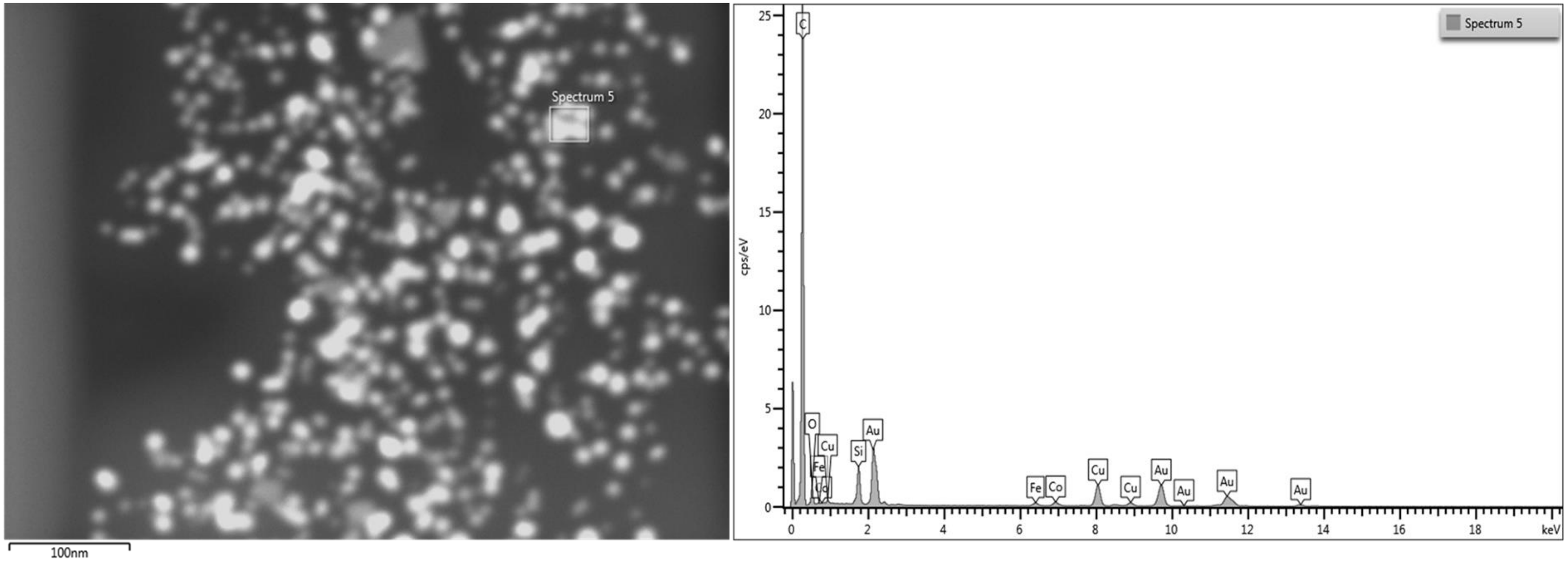

Figure 2: EDS spectra of HO-MeOH – GA AuNPs

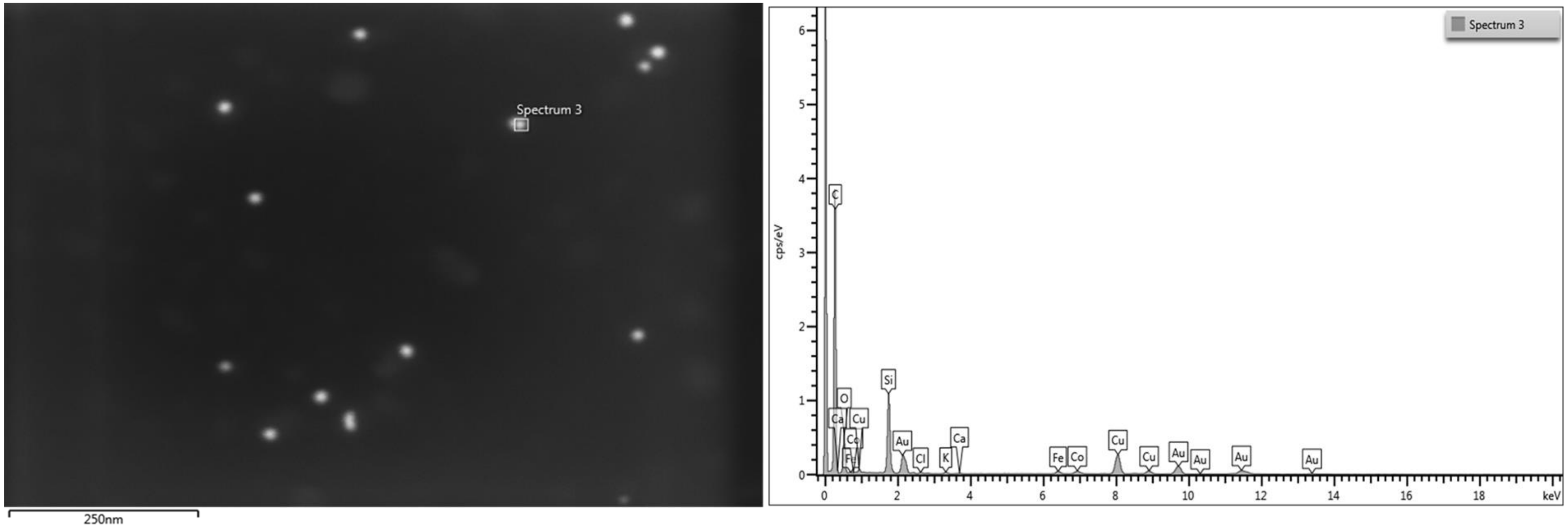

Figure 3: EDS spectra of HO-Powder + GA AuNPs

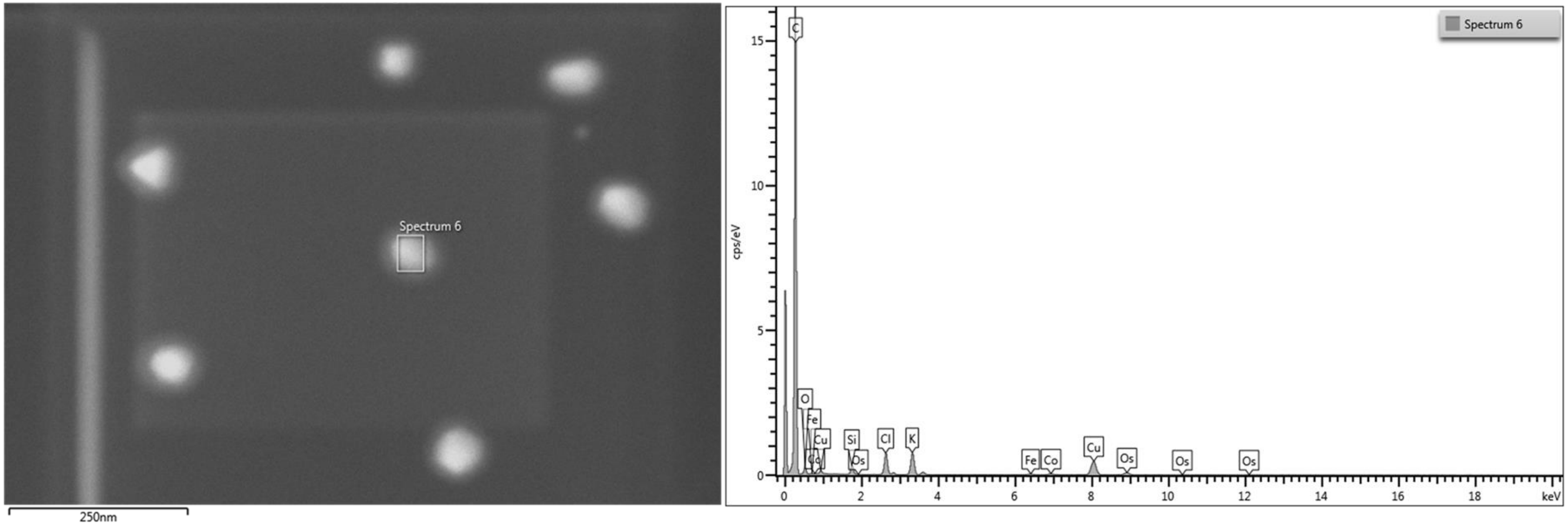

Figure 4: EDS spectra of HO-Powder - GA AuNPs

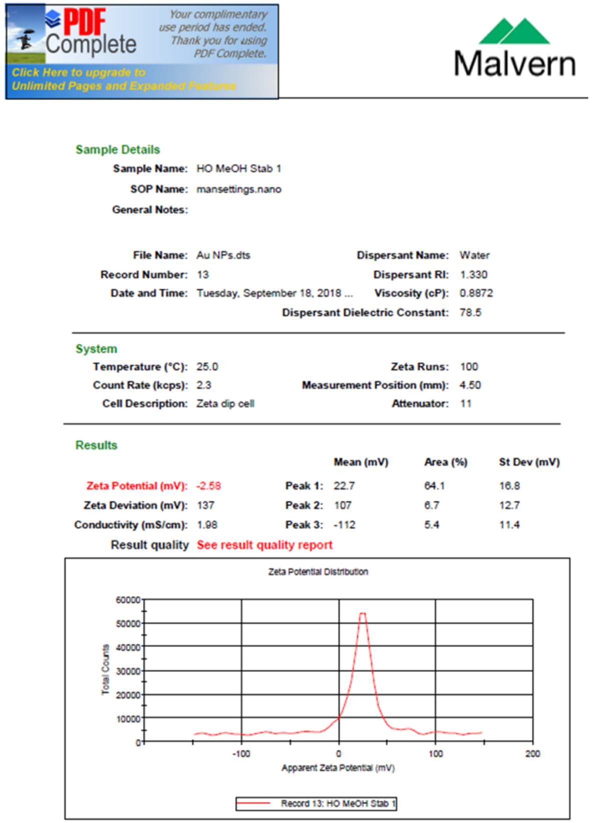
PDF Complete
Your complimentary use period has ended. Thank you for using PDF Complete.
Click Here to upgrade to Unlimited Pages and Expanded Features

Malvern

Sample Details

Sample Name: HO MeOH Stab 1
SOP Name: mansettings.nano
General Notes:

**File Name:**	Au NPs.dts	**Dispersant Name:**	Water
**Record Number:**	13	**Dispersant RI:**	1.330
**Date and Time:**	Tuesday, September 18, 2018 ...	**Viscosity (cP):**	0.8872
		**Dispersant Dielectric Constant:**	78.5

System

**Temperature (°C):**	25.0	**Zeta Runs:**	100
**Count Rate (kcps):**	2.3	**Measurement Position (mm):**	4.50
**Cell Description:**	Zeta dip cell	**Attenuator:**	11

Results

			Mean (mV)	Area (%)	St Dev (mV)
**Zeta Potential (mV):**	-2.58	**Peak 1:**	22.7	64.1	16.8
**Zeta Deviation (mV):**	137	**Peak 2:**	107	6.7	12.7
**Conductivity (mS/cm):**	1.98	**Peak 3:**	-112	5.4	11.4

Result quality See result quality report

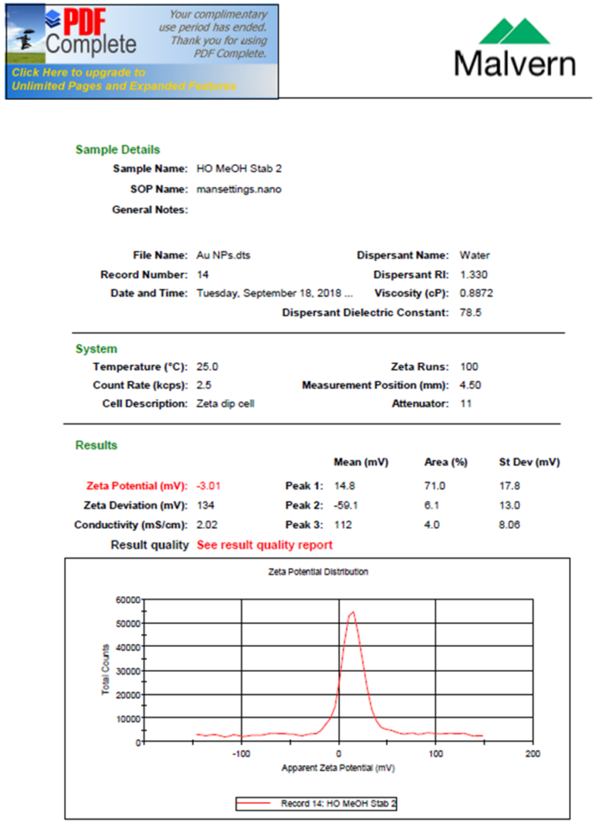
PDF Complete
Your complimentary use period has ended. Thank you for using PDF Complete.
Click Here to upgrade to Unlimited Pages and Expanded Features

Malvern

Sample Details

Sample Name: HO MeOH Stab 2
SOP Name: mansettings.nano
General Notes:

**File Name:**	Au NPs.dts	**Dispersant Name:**	Water
**Record Number:**	14	**Dispersant RI:**	1.330
**Date and Time:**	Tuesday, September 18, 2018 ...	**Viscosity (cP):**	0.8872
		**Dispersant Dielectric Constant:**	78.5

System

**Temperature (°C):**	25.0	**Zeta Runs:**	100
**Count Rate (kcps):**	2.5	**Measurement Position (mm):**	4.50
**Cell Description:**	Zeta dip cell	**Attenuator:**	11

Results

			Mean (mV)	Area (%)	St Dev (mV)
**Zeta Potential (mV):**	-3.01	**Peak 1:**	14.8	71.0	17.8
**Zeta Deviation (mV):**	134	**Peak 2:**	-59.1	6.1	13.0
**Conductivity (mS/cm):**	2.02	**Peak 3:**	112	4.0	8.06

Result quality See result quality report

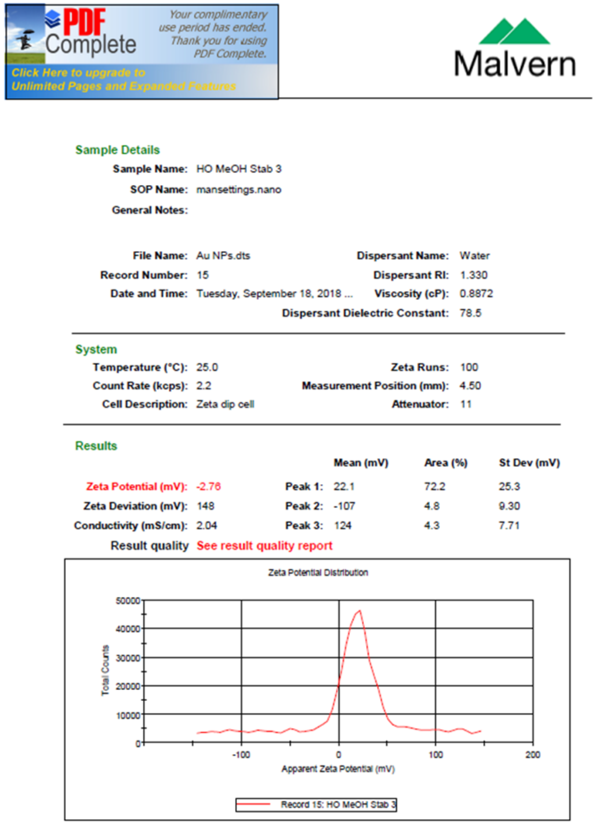
PDF Complete
Your complimentary use period has ended. Thank you for using PDF Complete.
Click Here to upgrade to Unlimited Pages and Expanded Features

Malvern

Sample Details

Sample Name: HO MeOH Stab 3
SOP Name: mansettings.nano
General Notes:

**File Name:**	Au NPs.dts	**Dispersant Name:**	Water
**Record Number:**	15	**Dispersant RI:**	1.330
**Date and Time:**	Tuesday, September 18, 2018 ...	**Viscosity (cP):**	0.8872
		**Dispersant Dielectric Constant:**	78.5

System

**Temperature (°C):**	25.0	**Zeta Runs:**	100
**Count Rate (kcps):**	2.2	**Measurement Position (mm):**	4.50
**Cell Description:**	Zeta dip cell	**Attenuator:**	11

Results

			Mean (mV)	Area (%)	St Dev (mV)
**Zeta Potential (mV):**	-2.76	**Peak 1:**	22.1	72.2	25.3
**Zeta Deviation (mV):**	148	**Peak 2:**	-107	4.8	9.30
**Conductivity (mS/cm):**	2.04	**Peak 3:**	124	4.3	7.71

Result quality See result quality report

Figure 5: Zeta potential charges of 3 runs of HO-MEOH + GA AuNPs

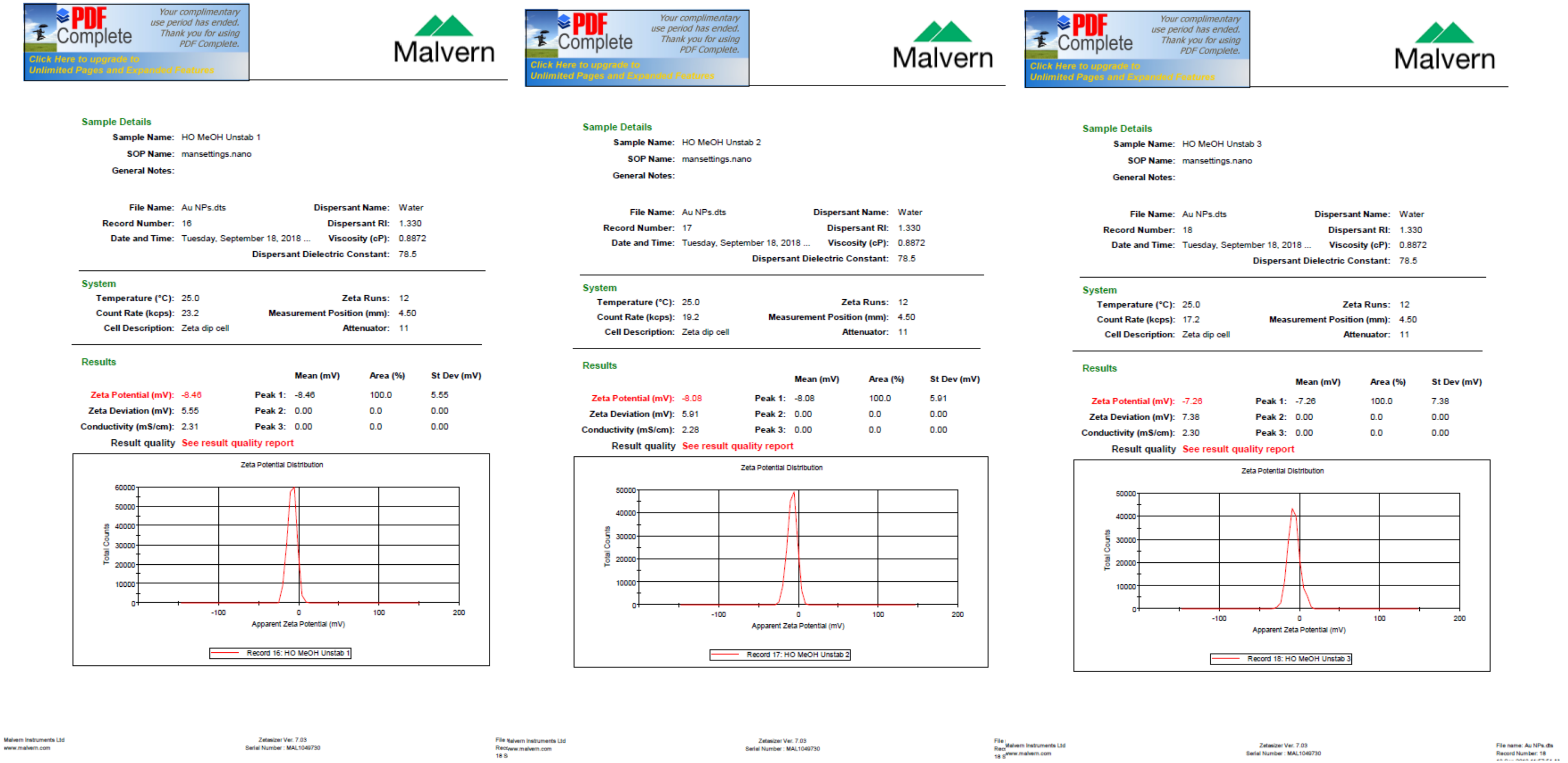

Figure 6: Zeta potential charges of 3 runs of HO-MEOH - GA AuNPs

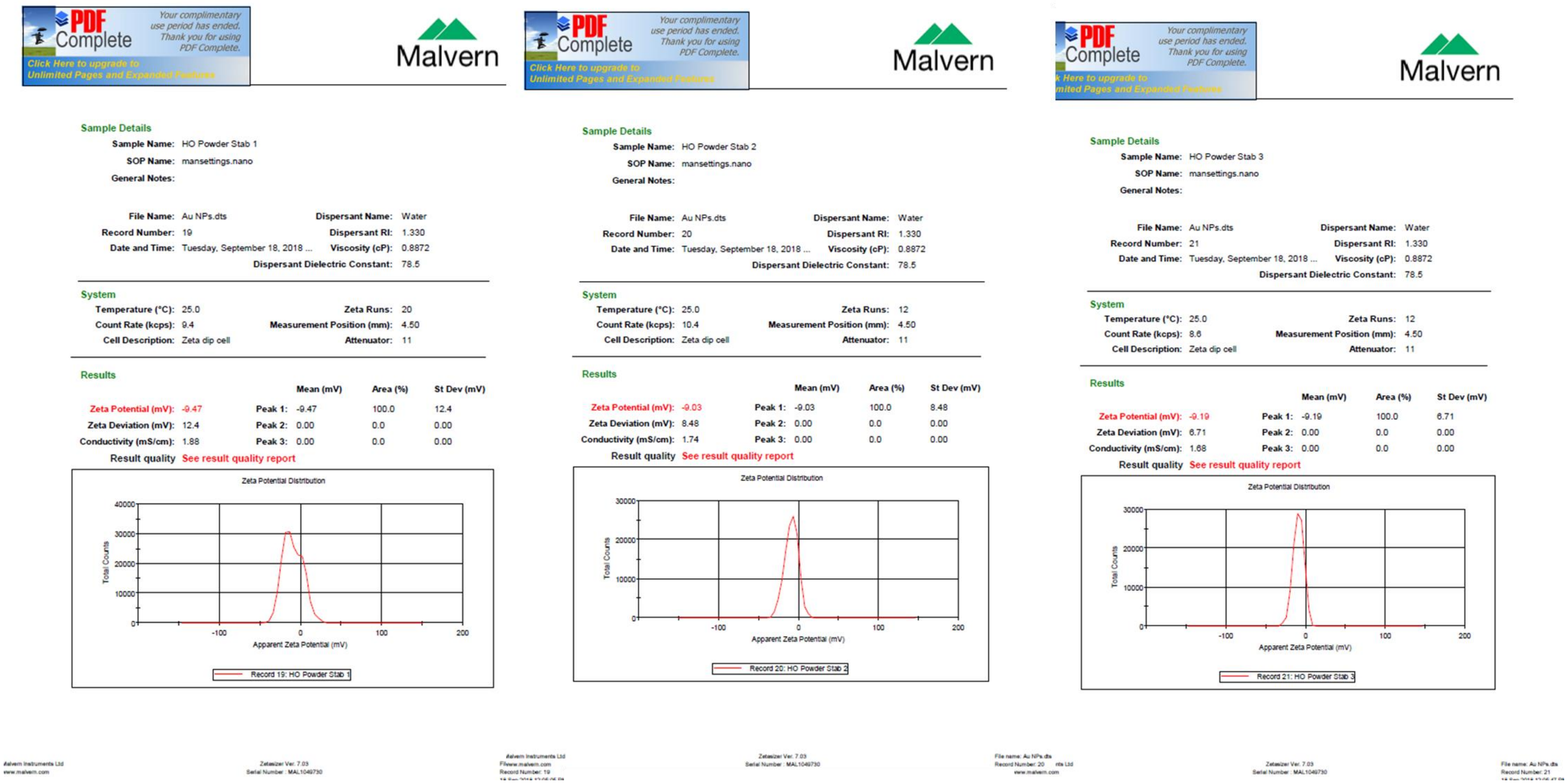

Malvern

Sample Details

Sample Name: HO Powder Stab 1
SOP Name: mansettings.nano
General Notes:

File Name:	Au NPs.dts	Dispersant Name:	Water
Record Number:	19	Dispersant RI:	1.330
Date and Time:	Tuesday, September 18, 2018 ...	Viscosity (cP):	0.8872
		Dispersant Dielectric Constant:	78.5

System

Temperature (°C):	25.0	Zeta Runs:	20
Count Rate (kcps):	9.4	Measurement Position (mm):	4.50
Cell Description:	Zeta dip cell	Attenuator:	11

Results

			Mean (mV)	Area (%)	St Dev (mV)
Zeta Potential (mV):	-9.47	Peak 1:	-9.47	100.0	12.4
Zeta Deviation (mV):	12.4	Peak 2:	0.00	0.0	0.00
Conductivity (mS/cm):	1.88	Peak 3:	0.00	0.0	0.00

Result quality: See result quality report

Malvern

Sample Details

Sample Name: HO Powder Stab 2
SOP Name: mansettings.nano
General Notes:

File Name:	Au NPs.dts	Dispersant Name:	Water
Record Number:	20	Dispersant RI:	1.330
Date and Time:	Tuesday, September 18, 2018 ...	Viscosity (cP):	0.8872
		Dispersant Dielectric Constant:	78.5

System

Temperature (°C):	25.0	Zeta Runs:	12
Count Rate (kcps):	10.4	Measurement Position (mm):	4.50
Cell Description:	Zeta dip cell	Attenuator:	11

Results

			Mean (mV)	Area (%)	St Dev (mV)
Zeta Potential (mV):	-9.03	Peak 1:	-9.03	100.0	8.48
Zeta Deviation (mV):	8.48	Peak 2:	0.00	0.0	0.00
Conductivity (mS/cm):	1.74	Peak 3:	0.00	0.0	0.00

Result quality: See result quality report

Malvern

Sample Details

Sample Name: HO Powder Stab 3
SOP Name: mansettings.nano
General Notes:

File Name:	Au NPs.dts	Dispersant Name:	Water
Record Number:	21	Dispersant RI:	1.330
Date and Time:	Tuesday, September 18, 2018 ...	Viscosity (cP):	0.8872
		Dispersant Dielectric Constant:	78.5

System

Temperature (°C):	25.0	Zeta Runs:	12
Count Rate (kcps):	8.6	Measurement Position (mm):	4.50
Cell Description:	Zeta dip cell	Attenuator:	11

Results

			Mean (mV)	Area (%)	St Dev (mV)
Zeta Potential (mV):	-9.19	Peak 1:	-9.19	100.0	6.71
Zeta Deviation (mV):	6.71	Peak 2:	0.00	0.0	0.00
Conductivity (mS/cm):	1.68	Peak 3:	0.00	0.0	0.00

Result quality: See result quality report

Figure 7: Zeta potential charges of 3 runs of Ho-Powder + GA AuNPs

PDF Complete
Your complimentary use period has ended. Thank you for using PDF Complete.
Click Here to upgrade to Unlimited Pages and Expanded Features

Malvern

Sample Details

Sample Name:	HO Powder Unstab 1
SOP Name:	mansettings.nano
General Notes:	

File Name:	Au NPs.dts	Dispersant Name:	Water
Record Number:	22	Dispersant RI:	1.330
Date and Time:	Tuesday, September 18, 2018 ...	Viscosity (cP):	0.8872
		Dispersant Dielectric Constant:	78.5

System

Temperature (°C):	25.0	Zeta Runs:	28
Count Rate (kcps):	6.4	Measurement Position (mm):	4.50
Cell Description:	Zeta dip cell	Attenuator:	11

Results

			Mean (mV)	Area (%)	St Dev (mV)
Zeta Potential (mV):	-4.14	Peak 1:	-4.14	100.0	6.58
Zeta Deviation (mV):	6.58	Peak 2:	0.00	0.0	0.00
Conductivity (mS/cm):	2.01	Peak 3:	0.00	0.0	0.00
Result quality	See result quality report				

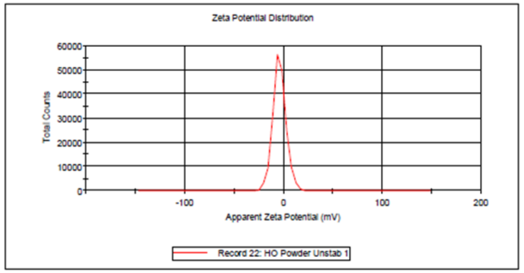

PDF Complete
Your complimentary use period has ended. Thank you for using PDF Complete.
Click Here to upgrade to Unlimited Pages and Expanded Features

Malvern

Sample Details

Sample Name:	HO Powder Unstab 2
SOP Name:	mansettings.nano
General Notes:	

File Name:	Au NPs.dts	Dispersant Name:	Water
Record Number:	23	Dispersant RI:	1.330
Date and Time:	Tuesday, September 18, 2018 ...	Viscosity (cP):	0.8872
		Dispersant Dielectric Constant:	78.5

System

Temperature (°C):	25.0	Zeta Runs:	78
Count Rate (kcps):	7.8	Measurement Position (mm):	4.50
Cell Description:	Zeta dip cell	Attenuator:	11

Results

			Mean (mV)	Area (%)	St Dev (mV)
Zeta Potential (mV):	-4.22	Peak 1:	-4.22	100.0	7.39
Zeta Deviation (mV):	7.39	Peak 2:	0.00	0.0	0.00
Conductivity (mS/cm):	2.03	Peak 3:	0.00	0.0	0.00
Result quality	See result quality report				

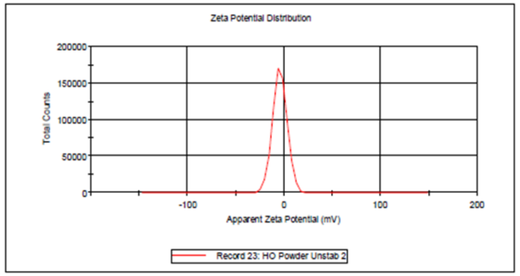

PDF Complete
Your complimentary use period has ended. Thank you for using PDF Complete.
Click Here to upgrade to Unlimited Pages and Expanded Features

Malvern

Sample Details

Sample Name:	HO Powder Unstab 3
SOP Name:	mansettings.nano
General Notes:	

File Name:	Au NPs.dts	Dispersant Name:	Water
Record Number:	24	Dispersant RI:	1.330
Date and Time:	Tuesday, September 18, 2018 ...	Viscosity (cP):	0.8872
		Dispersant Dielectric Constant:	78.5

System

Temperature (°C):	25.0	Zeta Runs:	12
Count Rate (kcps):	5.5	Measurement Position (mm):	4.50
Cell Description:	Zeta dip cell	Attenuator:	11

Results

			Mean (mV)	Area (%)	St Dev (mV)
Zeta Potential (mV):	-3.54	Peak 1:	-3.54	100.0	9.26
Zeta Deviation (mV):	9.26	Peak 2:	0.00	0.0	0.00
Conductivity (mS/cm):	2.00	Peak 3:	0.00	0.0	0.00
Result quality	See result quality report				

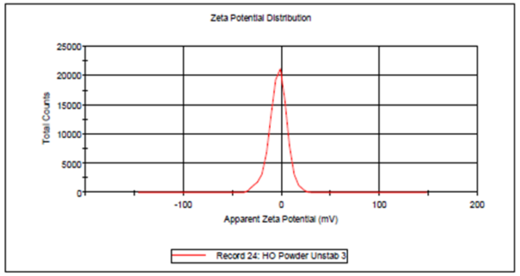

Figure 8: Zeta potential charges of 3 runs of Ho-Powder - GA AuNPs

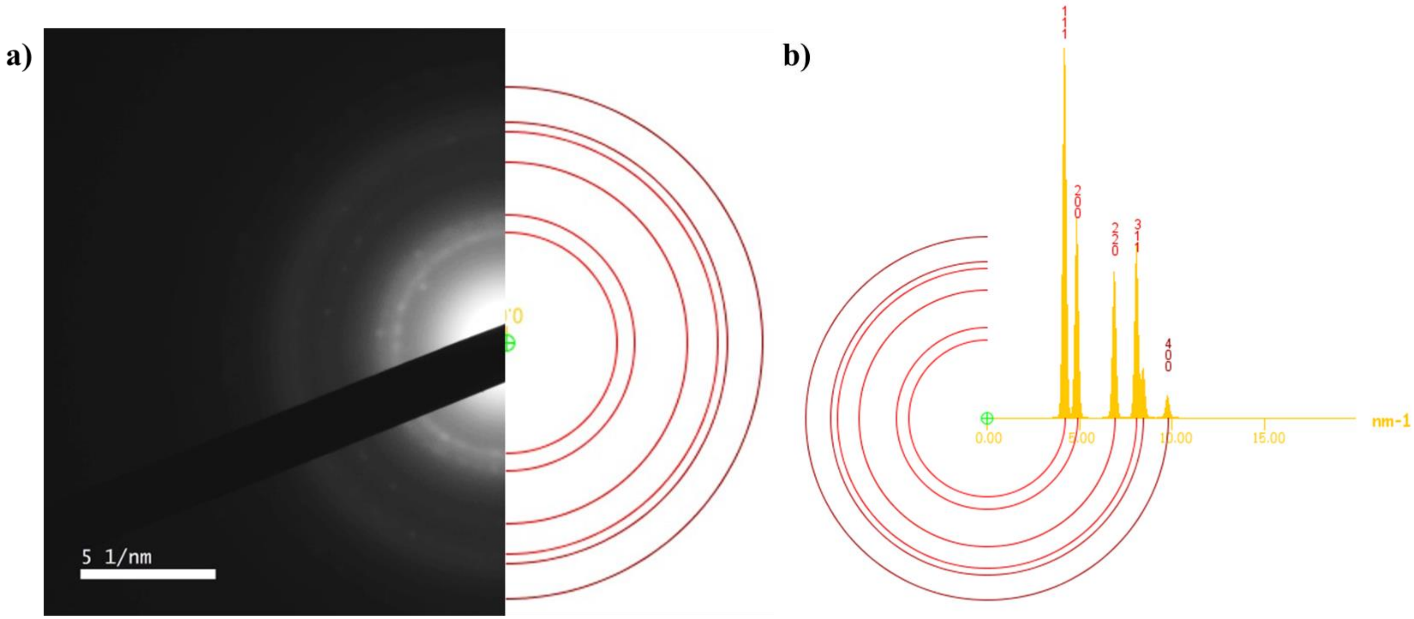

Figure 9: a) Sherrer ring patterns matching b) the gold standard for face centred cubic crystal structure
